# Mechanistic advances in osteoporosis and anti‐osteoporosis therapies

**DOI:** 10.1002/mco2.244

**Published:** 2023-05-11

**Authors:** Haiwei Wang, Yuchuan Luo, Haisheng Wang, Feifei Li, Fanyuan Yu, Ling Ye

**Affiliations:** ^1^ State Key Laboratory of Oral Diseases & National Clinical Research Center for Oral Diseases West China Hospital of Stomatology Sichuan University Chengdu China; ^2^ Department of Endodontics West China Hospital of Stomatology Sichuan University Chengdu China

**Keywords:** anti‐osteoporosis therapies, biomedical mechanism, osteoporosis, pathogenesis, small molecular interventions

## Abstract

Osteoporosis is a type of bone loss disease characterized by a reduction in bone mass and microarchitectural deterioration of bone tissue. With the intensification of global aging, this disease is now regarded as one of the major public health problems that often leads to unbearable pain, risk of bone fractures, and even death, causing an enormous burden at both the human and socioeconomic layers. Classic anti‐osteoporosis pharmacological options include anti‐resorptive and anabolic agents, whose ability to improve bone mineral density and resist bone fracture is being gradually confirmed. However, long‐term or high‐frequency use of these drugs may bring some side effects and adverse reactions. Therefore, an increasing number of studies are devoted to finding new pathogenesis or potential therapeutic targets of osteoporosis, and it is of great importance to comprehensively recognize osteoporosis and develop viable and efficient therapeutic approaches. In this study, we systematically reviewed literatures and clinical evidences to both mechanistically and clinically demonstrate the state‐of‐art advances in osteoporosis. This work will endow readers with the mechanistical advances and clinical knowledge of osteoporosis and furthermore present the most updated anti‐osteoporosis therapies.

## Graphical abstract

Osteoporosis, a type of bone loss disease, causes enormous burden at both the human and socioeconomic layer. In this study, we systematically reviewed according literatures and clinical evidences to both mechanistically and clinically demonstrate the state‐of‐art advances on osteoporosis, and furthermore present the most updated anti‐osteoporosis therapies.

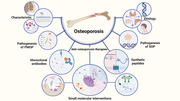

## 1 INTRODUCTION

**FIGURE 1:**
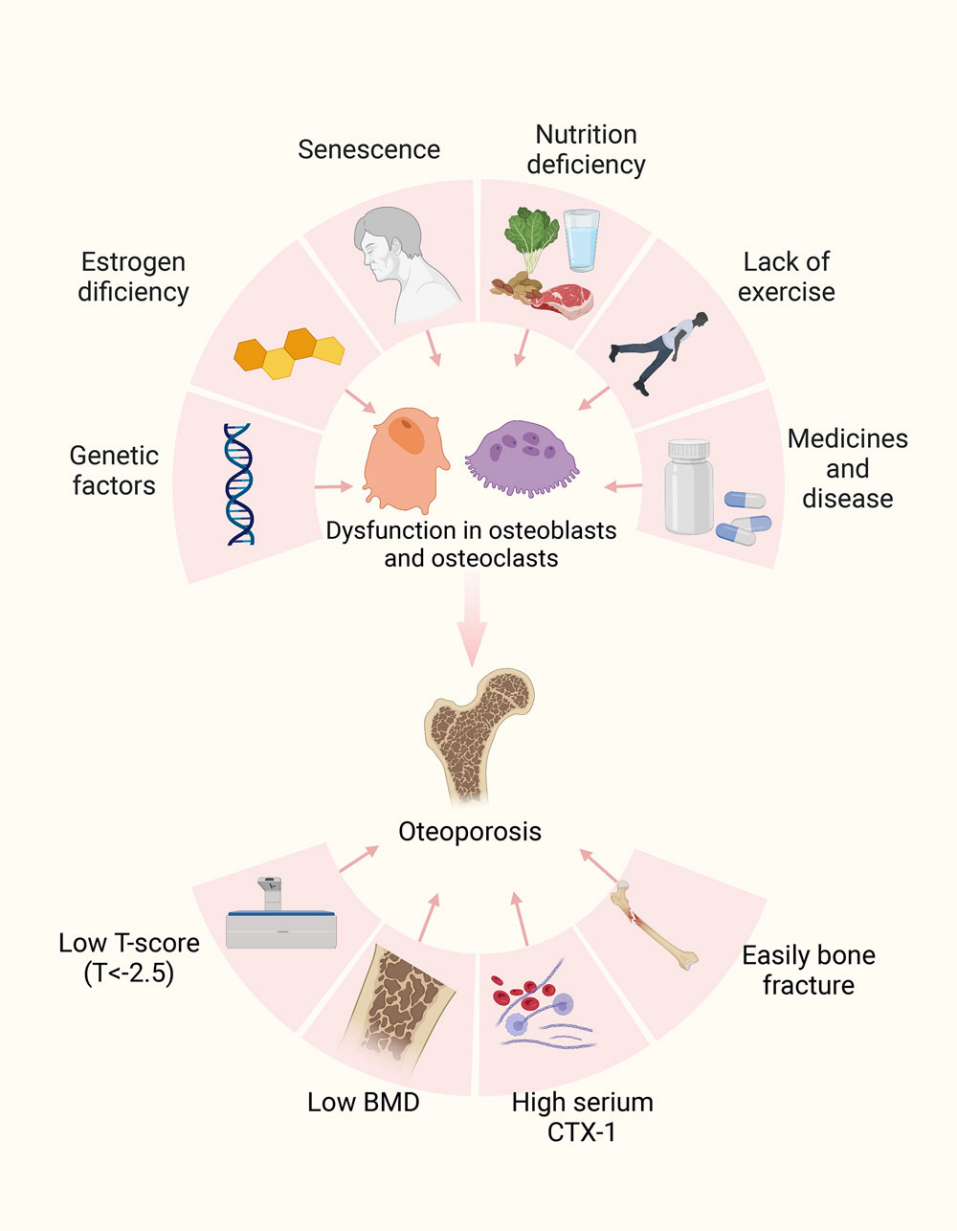
The overview of etiologies and characteristics of osteoporosis. Genetic factors, hormone deficiency and senescence, etc., can lead to the dysfunction of osteoblasts and osteoclasts thus causing osteoporosis. Osteoporosis can increase the risk of fractures in different body parts, along with changes in serological indicators and imaging parameters. BMD, bone mineral density.

Osteoporosis is a complex disease that is affected by many factors such as inheritance, sex, age, and environment (Figure 1). According to the initiation risks, osteoporosis can be divided into primary osteoporosis and secondary osteoporosis. Primary osteoporosis is usually the result of age‐related changes and is likely to occur in the elderly, including postmenopausal osteoporosis (PMOP), senile osteoporosis (SOP), and idiopathic osteoporosis (IOP). Secondary osteoporosis is caused by unspecific diseases and drugs affecting bone metabolism. Increased bone fragility and fracture susceptibility caused by osteoporosis can lead to unbearable pain, mobility impairments, and even death. With the increasing pressure of global aging population, osteoporosis may become a new worldwide challenge, causing an enormous burden at both the human and socioeconomic layer. Statistics show that nearly one‐third of women and one‐fifth of men in the world are suffering or will suffer from osteoporosis.^1^, ^2^

The complete structure and sound function of bone tissue depend on the bone homeostasis maintained by osteoblasts and osteoclasts. After reaching the peak bone mass before age 35 years, calcium, phosphorus, and other matrix elements in the skeleton will be lost to different degrees.^3^, ^4^ The imbalance of bone remodeling continues to accumulate, resulting in the reduction of bone mass and microarchitectural deterioration of bone tissue, ultimately leading to osteoporosis. Menopause and senescence affect the balance between osteoclasts and osteoblasts in different ways, thus presenting different characteristics of bone turnover rate.

Classic anti‐osteoporosis therapies include anti‐resorptive and anabolic agents. Although a large number of drugs have been developed to prevent fractures, many patients still do not receive adequate treatment due to poor compliance and large side effects.^5^ In this study, we divided anti‐osteoporosis therapy into three categories: small molecular interventions, monoclonal antibodies, and synthetic peptides, according to the source, mode of action, and characteristics of drugs. Starting from the etiology and pathogenesis of primary osteoporosis, we systematically reviewed new therapeutic strategies or new clinical evidence on the basis of existing anti‐osteoporosis therapies, hoping to endow readers with the mechanistical advances and clinical knowledge of osteoporosis.

## 2 ETIOLOGY OF OSTEOPOROSIS

### 2.1 Genetic factors

Osteoporosis has strong genetic susceptibility. Family studies show that a significant amount of the variance in peak bone mass is genetically determined.^6^ Twins Study revealed that genetic factors account for 25%–45% of the variation in age‐related bone loss.^7^ Genome‐wide association studies have identified over 200 osteoporosis susceptibility loci, including *COL1A1*, *COLIAI2*, *LRP5*, *SOST*, etc., but the clear structure, number, distribution, and size remain largely unknown.^8^ Among them, *DAAM2* used to be predicted to play a role in canonical Wnt signaling.^9^ Further study showed that in the femur of mice with *Daam2* knockdown, the porosity of cortical bone increases and the structure of bone trabecula is impaired, resulting in a significant decrease in bone strength.^10^

### 2.2 Endocrine factors

A number of hormones contribute to bone metabolism, including estrogen, testosterone, calcitonin, and parathyroid hormone (PTH). Changes in estrogen levels in women during pregnancy or after menopause can lead to bone loss and risk of fractures.^11^, ^12^ Similarly, severe gonadal steroid deficiency induces bone loss in adult men, but the impact of testosterone level change is relatively small.^13^ PTH has a two‐way effect on bone regulation. Chronic deficiency of PTH in patients with hypoparathyroidism can lead to abnormality of bone microstructure, reduction in bone remodeling, and increased risk of vertebral fracture.^14^, ^15^ In hyperparathyroidism, continuous exposure to high levels of PTH causes increased bone absorption, and even mild symptoms can reduce bone mineral density (BMD) and cause fragility fractures.^16^ Although the function of calcitonin is weak in physiological state and there is no correlation between basal calcitonin level or calcitonin reserve and change in BMD,^17^ calcitonin together with PTH can maintain normal calcium and phosphorus metabolism, which is vital for maintaining bone mass.^18^ Meanwhile, excessive glucocorticoids due to self‐reason (hypercortisolism) or long‐term medication can increase the risk of vertebral fractures and bone loss.^18^, ^19^ However, the dose and severity of glucocorticoids are not positively correlated. Similarly, hypermetabolism caused by hyperthyroidism can also lead to osteoporosis.^20^

### 2.3 Senescence

Osteoporosis is one of the most common diseases associated with senescence. More than half of adults over 50 years old suffer from osteoporosis in the USA.^2^ One of the main causes of SOP is a decrease in hormone levels. Age‐related inactivation of hormone‐binding globulin can lead to testosterone and estrogen inactivation.^21^ A further consequence of senescence is a reduction in osteoblast and osteoclast synthesis and secretion ability, which will slow down the rate of bone reconstruction.

### 2.4 Nutritional status

Calcium, phosphorus, and magnesium are the main elements of bone, and the deficiency or proportion change of these elements may lead to bone synthesis disorder and increased bone loss. Adequate calcium and dietary protein intake can reduce fracture risk.^22^ Conversely, insufficient nutritional calcium and vitamin D can cause rickets or osteomalacia in children or adolescents and osteoporosis in adults.^23^ Calcium supplements to maintain calcium balance have now been regarded as a standardized basic means to prevent and treat osteoporosis.

### 2.5 Lifestyle

Smoking has been identified as an explicit risk factor for osteoporosis. Smoking is associated with reduced bone mass and increased bone loss, while quitting smoking has been clearly shown to improve BMD and reduce fracture risk.^24^ The effect of alcohol on bone depends on the dosage. Although light (less than 15 g/day) to moderate (less than 30 g/day) drinking generally lowers the risk of fractures,^25^ consuming more alcohol will lead to a continuous increase in osteoporotic fracture risk.^26^

A prospective study suggests that sarcopenia has a higher risk of developing osteoporosis.^27^ Moderate physical activity can exert mechanical stimulation on bones and strengthen muscle function, which is considered as a bone protection factor. Mechanical loading above daily activity can improve BMD.^28^ Many studies have demonstrated that exercise can improve physical function, enhance the quality of life, and reduce pain in PMOP patients.^29^ A meta‐analysis showed that different kinds of exercise have various effects on the BMD of different parts of skeleton.^30^

Being aware of etiologies of osteoporosis can help clinicians prevent the occurrence of osteoporosis, and further insights in the molecular mechanisms contribute to the development of both prevention and intervention treatments. As a result, we will have a brief review of advances in molecular mechanisms of osteoporosis to benefit future clinical work.

## 3 PATHOGENESIS OF PMOP

Approximately half of women will once have a fracture after the age of 50 years due to the decrease in estrogen.^31^ In fact, women have higher rates of osteoporosis than men at any age because of estrogen deficiency.^32^ Estrogen has a significant impact on maintaining the homeostasis of the endocrine system, cardiovascular system, metabolic system, and bone development. Estrogen deficiency induces bone loss in both cancellous and cortical.^33^ Ovarian aging results in a higher risk of osteoporosis in women.^34^, ^35^ Even in men, estrogen remains the major regulator of the bone.^36^ Having precise knowledge on the role of estrogen in bone metabolism is the foundation of managing PMOP. Estrogen acts in a “two‐step” way. First, estrogen binds to the estrogen receptors (ERs) in the cytoplasm, and the ERs then dimerize and translocate to the nucleus.^37^ Estrogen passively passes through the cell and nuclear membranes and then combines with the receptor. The complex of ligand and receptor binds to specific sequences in the regulatory region of the target genes, and these sequences of DNA are known as estrogen response elements (EREs).^38^

**FIGURE 2:**
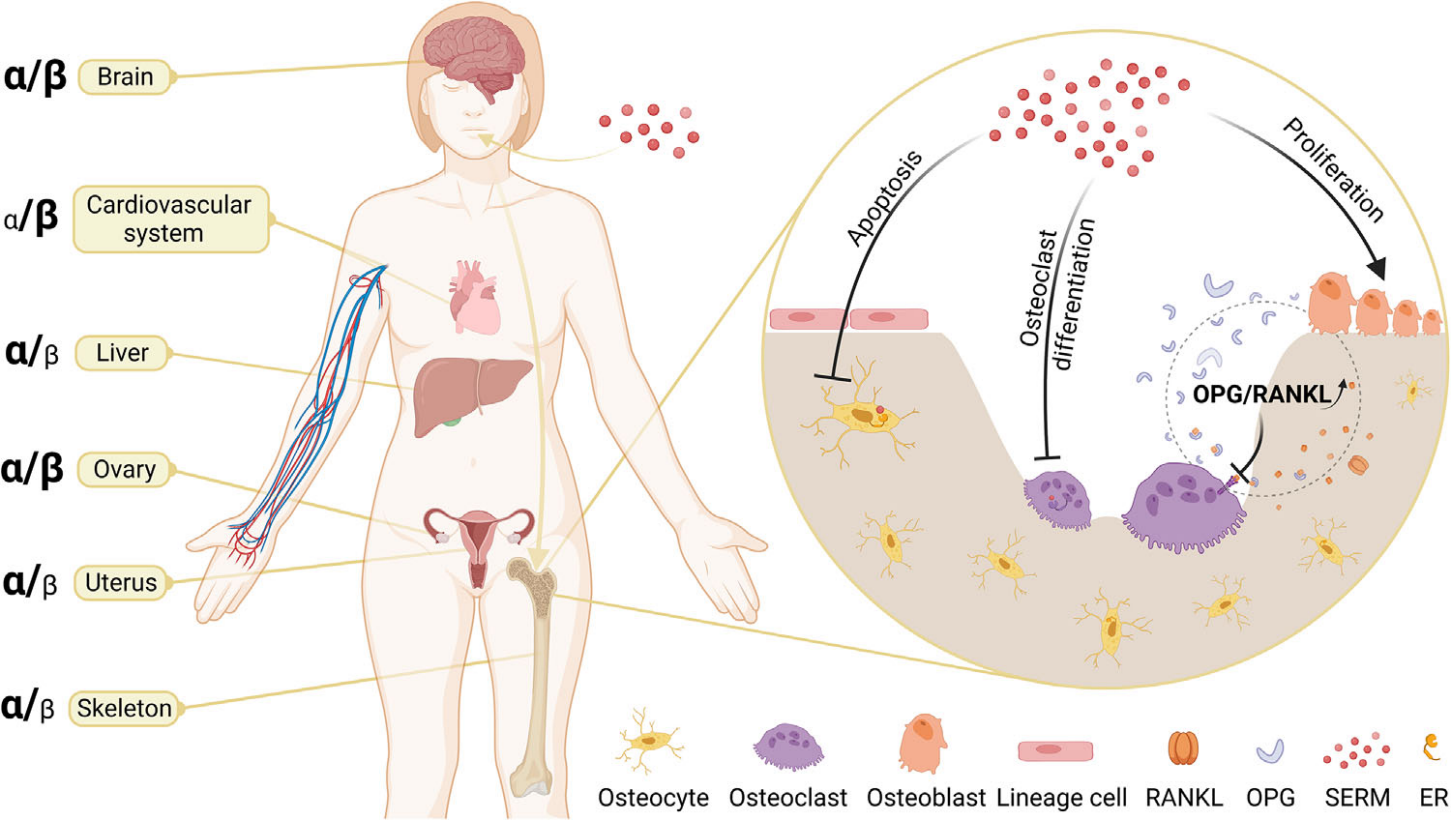
The mechanism of selective estrogen receptor modulators (SERMs) on bone homeostasis. The main estrogen receptor types (ERα or ERβ) distributed in different tissues and organs are different. SERMs can selectively activate ERs in the skeleton after oral administration, regulate osteoblasts, osteoclasts, and osteocytes, and upregulate the ratio of osteoprotegerin (OPG) to receptor activator of nuclear kappa‐B ligand (RANKL).

There are two types of ERs, alpha (ERα) and beta (ERβ). They have different affinities to estrogen and different distributions^39^ (Figure 2). ERs belong to the steroid/thyroid hormone superfamily of nuclear receptors. All members are composed of three functional domains, which are independent of each other but interact. The DNA‐binding domains are different between ERα and ERβ. Besides, the ligand‐binding sites of ERα and ERβ have slight differences, which enables the development of selective estrogen receptor modulators (SERMs)^40^ (Figure 2).

Postmenopause can affect estrogen by decreasing the level and changing the concentration of different estrogen. There are three major forms of physiological estrogens in females: estrone (E1), estradiol (E2 or 17β‐estradiol), and estriol (E3). In postmenopause, serum E2 levels decrease by 85%–90% and serum E1 levels decrease by 65%–75% from mean premenopausal levels.^41^ The decline in estrogen after menopause can affect bone mass in many ways, and we will introduce these effects, which may become a guidance of medical choices.^41^

The long list of possible targets of ER includes cytokines, such as interleukin (IL)‐1,^42^, ^43^ IL‐17,^44^, ^45^ IL‐6,^43^, ^46^ IL‐7,^47^, ^48^, ^49^ and tumor necrosis factor‐alpha (TNF‐α)^43^, ^50^ (Table 1). Cells such as T and B lymphocytes, macrophages, and dendritic cells are affected by estrogen, too.^51^

**TABLE 1:** List of pathogeneses of postmenopausal osteoporosis (PMOP) and approaches these pathogeneses affect osteoblasts and osteoclasts.

Pathogenesis of PMOP	Ways to have impacts	Influences on osteoblasts or osteoclasts	References
Inflammation due to estrogen deficiency	Interleukin‐1	Increasing osteoclasts formation	^42^
		Promoting osteoclasts survival	^43^
	Interleukin‐17	Stimulating osteoblast and osteoclasts differentiation	^44^
		Stimulating expression of RANK and receptor of M‐CSF	^45^
	Interleukin‐6	Indirectly stimulating osteoclasts activity via stimulating expression of interleukin‐1	^43^
		Stimulating osteoclasts differentiation in a RANKL‐independent way	^46^
	Interleukin‐7	Indirectly stimulating osteoclasts activity via stimulating expression of TNF‐α and RANKL	^47^, ^48^
		Inhibiting osteoblastic bone formation.	^49^
	TNF‐α	Stimulating RANKL expressing	^43^
		Recruiting osteoclasts	
		Stimulating osteoclasts differentiation in a RANKL‐independent way	^50^
Estrogen deficiency	RANKL and M‐CSF	Inhibiting osteoclasts differentiation	^52^
	Fas/FasL system	Leading to osteoclasts apoptosis	^53^
	Promoting autophagy	Prolonging the survival of osteoblasts	^54^
	Upregulating BMP‐4	Prompting the synthesis function of osteoblasts	^55^
	Increasing in β1‐integrin expression	Augmenting shear stress responsiveness	^56^

Abbreviations: RANK, receptor activator of nuclear kappa‐B; RANKL, RANK ligand; TNF‐α, tumor necrosis factor‐alpha.

### 3.1 Estrogen deficiency directly related to osteoblast and osteoclast activity

Receptor activator of nuclear factor‐kappa‐b ligand (RANKL) is the crucial molecule needed for osteoclast development from myeloid precursors. Macrophage colony‐stimulating factor (M‐CSF)/RANKL signaling stimulates absorption activity. Estrogen blocks RANKL/M‐CSF‐induced activator protein‐1‐dependent transcription, thus restraining RANKL‐induced osteoclast differentiation.^52^ Estrogen inhibits RANKL‐stimulated osteoclastic differentiation of monocytes by inducing ERα binding to BCAR1, a scaffolding protein.^57^ However, mechanisms by which estrogen interacts with osteoclasts via RANKL signaling remain ambiguous. Other mechanisms have also been proposed. Binding of estrogen and ERα leads to osteoclast apoptosis via Fas/FasL system‐mediated apoptotic induction.^53^

Comparing protective efficiency in LYN (a kind of intracellular membrane‐associated protein tyrosine kinase, which has a crucial function in signaling intermediaries) knockdown osteoclasts and control conditions shows the importance of LYN as a key mediator of the effect of estrogen on osteoclastogenesis.^58^ There are reports about osteoclast‐specific HIF1α inactivation antagonizes estrogen‐deficient bone loss, and the HIF1α was destabilized by estrogen treatment.^59^ Thus, HIF1α represents a promising therapeutic target in osteoporosis.

Furthermore, estrogen has an effect on osteoblasts. Estrogen can help prolong the survival of osteoblasts. Estrogen reduced apoptosis in differentiating osteoblasts by promoting autophagy, thus contributing to their longer lifespan.^54^ Estrogen has been shown to protect osteoblasts from apoptosis, and the protective effect may be mediated by heat shock protein 27.^60^

Estrogen can prompt the synthesis function of osteoblasts. Estrogen upregulates BMP‐4‐induced Smad1/5/8 phosphorylation in osteoblasts, and the effects can be reversed by the presence of ER antagonists.^55^ In addition, ERα in osteoblast progenitors expressing Osterix1 (Osx1) potentiates Wnt/β‐catenin signaling, thereby increasing the proliferation and differentiation of periosteal cells.^61^ Estrogen deficiency is not restricted to osteoclasts and bone resorption but also affects bone matrix composition and response of osteoblasts to mechanical stimulation.^62^ There are reports about estrogen augments shear stress responsiveness of signal transduction and gene expression in bone cells via ER‐mediated increases in β1‐integrin expression.^56^

### 3.2 Estrogen deficiency indirectly related to osteoblast and osteoclast activity

Estrogen also indirectly regulates osteoclasts.^44^, ^63^ Postmenopausal women often display a chronic low‐grade inflammatory phenotype with altered cytokine expression and immune cell profile.^44^ Estrogen deficiency in humans elevates the expression of RANKL in lymphocytes.^63^ B lymphocytopenia can be regarded as a characteristic of osteopetrosis, suggesting that B lymphopoiesis is regulated by osteoclast activity.^64^ CD22, shp‐1, bcl‐2, and vcam‐1 have been upregulated by estrogen via activating ERα and ERβ in B cells, and overexpression of CD22 and SHP‐1 in B cells decreases B‐cell receptor signaling.^65^ Factors affecting B‐cell function also affect osteoclasts and osteocytes, indicating a regulatory relationship between B lymphopoiesis, osteoclastogenesis, and osteoblastogenesis. Transcription factors required for B‐cell differentiation have unpredicted, pronounced, and non‐overlapping effects on osteoblast and osteoclast development.^66^

Human estrogen deficiency expands RANKL‐expressing T and B cells. Besides, T‐cell‐produced TNF‐α in postmenopause leads to bone loss.^67^ Loss of estrogen leads to inflammation that promotes osteoporosis. T cells and proinflammatory cytokines are closely connected with estrogen loss. E2 loss leads to chronic low‐grade production of the proinflammatory cytokines TNFα and IL‐17, both of which are important for osteoclast differentiation.^68^ Anti‐IL‐17 antibody preserved cortical bone parameters, bone biomechanical properties, and histomorphometry in animal models.^69^ TNFα can induce osteoclast differentiation RANKL‐independent manner, although limited. It also has a synergic effect on RANKL‐stimulated osteoclastogenesis.^50^, ^70^ Pro‐inflammatory cytokine IL‐1 causes activation of the inducible nitric oxide pathway in bone cells and induces bone loss. Recently, the cytokine IL‐6 has joined the form of cytokines as a bone‐reactive agent.^71^, ^72^ There have been reports identifying IL‐1β‐induced osteoclasts as a contributor to bone erosion in arthritis.^73^ At the same time, these inflammatory cytokines also have an effect on inflammatory cells, so the effect of estrogen is not a single line but a complex network. We list the cytokines that are regulated by estrogen and have effects on bone metabolism in order to have a clearer understanding of how estrogen regulates bone metabolism (Table 1).

Reactive oxygen species greatly influence the generation and survival of osteoclasts, osteoblasts, and osteocytes. Loss of estrogen or androgen decreases the defense against oxidative stress in bone, which explains the decrease in bone matrix associated with the acute loss of these hormones to some extent.^74^

### 3.3 Other influences of estrogen deficiency on bone metabolism

Estrogen can also have crosstalk with other hormones, which have effects on bone. Estrogen promotes calcitonin secretion and inhibits bone absorption, enhances the activity of hepatic 25‐hydroxylase and renal 1 alpha‐hydroxylase, increases the expression level of 1,25‐(OH)2D3, and promotes intestinal calcium absorption.^75^ In vitro culture experiments demonstrate that E2 and progesterone can stimulate PTH secretion by rapid, direct, and specific effects on parathyroid cells, which implies that in vivo PTH can also be affected by estrogen and disturbed in the postmenopausal period.^76^

An integrated 16S rRNA gene sequencing and liquid chromatography–mass spectrometry‐based metabolomics approach shed light on the possible relationship between BMD and gut microbiota/metabolite alterations in PMOP.^77^

## 4 PATHOGENESIS OF SOP

Senescence is involved in changes in biological phenomena of osteoblasts and osteocytes, lack of exercise, and nutrition intake. All of them disturb bone turnover, and we will review how senescence influences bone metabolism. SOP and PMOP are associated intimately but still have some differences.

### 4.1 Senescence in stem cells

Senescence is a complex process associated with various structural, functional, and metabolic changes. At the biological level, senescence results from the impact of the accumulation of a wide variety of molecular and cellular damage over time.

Mesenchymal stem cells (MSCs) are multipotential cells that can self‐renew and differentiate into various cell types, including osteogenic and adipogenic fates.^78^ Bone marrow mesenchymal stem cells (BMSCs) exhibit an age‐related lineage transformation from osteogenic to adipogenic fates, contributing to bone loss.^79^ As a result, the senescence of BMSCs directly influences skeletal formation by inhibiting osteogenesis. Human MSCs respond with a set of senescence programs to different stresses, including oxidative stress, damage stress, and chemical agents. The senescence programs of BMSCs include telomere dysfunction, DNA damage, irregular chromatin organization, and strong mitogenic pathway stimulation.^80^

When senescence‐related stimulation acts on stem cells, senescence‐related phenotypes are prompted by the retinoblastoma protein or p53 pathways. Then, the pathways activate the cyclin‐dependent kinase inhibitors p16 and p21, which are regarded as markers of senescence. It is worth noting that the pathways can interact with each other to induce senescence.^80^, ^81^, ^82^ Other pathways that mediate BMSC senescence are excavated. Researchers have found decreased autophagy in aged BMSCs compared with young BMSCs, and activation of autophagy could partially reverse the BMSC aging.^83^ This finding can be a potential therapy to oppose SOP. Depletion of SIRT3 leads to compromised nuclear integrity, loss of heterochromatin, and accelerated senescence in MSCs. The reintroduction of SIRT3 rescues the disorganized heterochromatin and the senescence phenotypes.^84^ In vitro research found an interaction between fibroblast growth factor 21 and senescence of MSCs, depletion of FGF21 enhanced the senescence of early‐passage MSCs, and overexpression of FGF21 in aged MSCs inhibited senescence.^85^

More than researches about alternations in phenotypes, mechanisms about functional influences of senescence have also been developed.^86^, ^87^ Senescence has an impact on BMSCs’ self‐renewal and osteogenic and intercellular communication.^88^, ^89^

FOXP1 expression level progressively declines with age. Conditional depletion of FOXP1 in BMSCs leads to premature aging characteristics and impairs MSC self‐renewal capacity in mice.^90^ As the common progenitors of adipocytes and osteoblasts, MSCs are delicately balanced; otherwise, the differentiation of osteoblasts and adipocytes will be tipped. Numerous in vitro studies have demonstrated that, although not strict, almost all adipogenic factors inhibit osteogenesis, and conversely, osteogenetic factors restrain adipogenesis.^91^, ^92^ Aside from familiar fate‐determining elements of osteogenesis, such as transforming growth factor‐β (TGFβ)/ bone morphogenetic proteins (BMPs, members included in TGFβ family), wingless‐integrated (WNT) signaling, etc. Long noncoding RNA (LncRNA) and extracellular vesicles (EVs) can also regulate MSCs’ fates during skeletal aging.^79^ How LncRNAs affect MSC is not precisely understood, but some studies have revealed some candidates of LncRNAs. For example, LncRNA‐MEG3 can upregulate the osteogenetic differentiation of MSCs. The changes between the secretomes of senescent BMSCs and young BMSCs may also lead to osteoporosis. Treatment with EVs from MSCs generated from human embryonic stem cells reduces senescence in vitro and in vivo.^93^ EVs from other stem cells can also rescue BMSC senescence. The umbilical cord‐produced MSC‐EVs contain abundant anti‐aging signals and rejuvenate senescing adult bone marrow‐derived MSCs.^94^

The functions of MSCs are not limited to their immense differentiation potential; they have a significant capacity in immunoregulation by inhibiting the immune response and exerting a function generally known as immune adjustment.^80^, ^95^ MSCs, can alter the frequency and function of memory lymphocytes, including Th17,^96^ follicular helper T (Tfh) cells,^97^ and gamma delta (γδ) T cells.^98^, ^99^ Dysregulation of T cells due to MSC senescence may be related to dysfunctions in bone metabolism, and more related research is needed in this territory.^100^, ^101^

Different lineages from MSCs have interactions, for example, osteolineage cells are believed to be a population linked to the regulation of hematopoietic stem cells.^102^ In an aged individual, both niches are interfered and this may contribute to osteoporosis.

Explorations in MSCs’ senescence modulation still cannot meet the need for clinical use, and there remain conundrums in inhibiting senescence in MSCs and clinical transformation.

### 4.2 Senescence in osteoblasts and osteoclasts

Age‐related osteoblast dysfunction related to changes in bone microenvironment and their own senescence. The age‐related impairment in MSCs leads to an impact on osteoblastic cell proliferation, and the underlying mechanisms are discussed above. However, the senescence impacts lifespan of osteoblasts. Enhancement of P53 leads to bone loss in mice. Likewise, genetic depletion of P53 will accumulate bone mass due to enhanced proliferation and reduced apoptosis.^103^, ^104^ The differentiation and function of osteoblasts are impaired. In rats, accumulation of preosteoblastic cells shows up with decreased number of mature osteoblasts with increasing age, suggesting that impaired osteoblast differentiation is a potential mechanism for age‐related impaired bone formation.^105^ A more recent in vitro study demonstrated that senescent cell conditioned medium disturbs osteoblast mineralization and enhances osteoclast progenitor survival.^106^

### 4.3 Hormone deficiency

Several hormones may play essential roles in bone homeostasis, including estrogen, testosterone, cortisol, PTH, and thyroid hormones. The inharmony in these hormones will impact the concentration of calcium/phosphate and bone homeostasis.^107^, ^108^ The impacts of estrogen deficiency have been discussed above and we specifically do not repeat here.

Parathyroid and thyroid hormones are both important systematic bone‐remolding modulators. Thyrotoxicosis is well known to cause severe osteoporosis and fracture. Subclinical thyroid disease will lead to bone loss and osteoporosis. Thyroid hormone can act in the skeleton directly by access to thyroid receptors.^109^ Significant and physiologic changes in thyroid parameters are observed during aging, and whether the changes tip the skeletal‐metabolism balance related to thyroid remains to be observed.^110^

The mechanisms by which PTH prompts osteoblast differentiation have not been thoroughly studied. However, in vitro studies have demonstrated that PTH induces osteoblast differentiation mainly via activation of the Wnt/β‐catenin pathway in osteoblastic MC3T3‐E1 cells.^111^ Intermittent PTH promotes osteoblast differentiation, in part, by its ability to promote exit from the cell cycle, to activate Wnt signaling in osteoblasts, and to inhibit the Wnt antagonist sclerostin in osteocytes.^112^ However, hypoparathyroidism is more prevalent in the younger population, which may related to surgery for thyroid disorders.^113^ But the studies in aging rats have revealed declines in PTH regulation of signal transduction pathways, which might relate to declines in the number of receptors.^114^

### 4.4 Exercise

Lack of exercise in old age can weaken bone and muscle parameters not limited to locomotor limbs but systematically.^115^ Local mechanical environment, such as bone strain, fluid shear flow, and electromagnetic fields within the bone influences osteoclasts convene to a particular location.^116^ The underlying theories have not been clear yet. But many studies have revealed the role of osteocytes in mechanical load bone remodeling. At the cellular level, mechanical load influences fluid flow in the osteocyte microenvironment. Investigating the molecular mechanisms of osteocyte mechanical–biological conversion is an urgent need.^117^ The finding of Piezo1, a mechanosensitive ion channel, explains the mechanism to some extent.^118^ Mechanical loading signals can transduce from osteocytes to osteoblasts via gap junction protein, Connexin43.^119^ Using the tooth‐movement model, researchers find that mechanical load elevates tooth movement and prompts the number of M1‐like macrophages.^120^ Mechanical loading can influence bone metabolism by having effects on osteocytes, osteoblasts, and osteoclasts.

Muscles and bones are considered as functional unities, and the degeneration in muscles will lead to bone loss. The potential molecular mechanisms are not limited in mechanical coupling theory but the secretory nature of bones and muscles can also affect each other. The secretory proteins included insulin‐like growth factor‐1 (IGF‐1), fibroblast growth factor‐2 (FGF‐2), IL‐6, IL‐15, myostatin, osteoglycin, family with sequence similarity 5,member C (FAM5C), Tmem119, and osteoactivin.^121^

Exercise also has an effect on endocrine. Exercise can influence serum PTH. Impact exercise training lowers the serum basal PTH levels and possibly enables greater difference between the basal PTH and transient exercise‐induced PTH peaks which lead to osteogenic effects.^122^ A 40‐min downhill exercise can prevent or mitigate PMOP in sedentary women by avoiding circadian PTH oversecretion.^123^

## 5 PATHOGENESIS OF IOP

In a study of the Mayo Clinic screening individuals aged 20–44 years, the incidence of IOP in this age group was only 0.4 cases per 100,000 person‐years, with no difference between genders. The cause of IOP is believed to be connected with genes. But which genes are related to IOP remains unclear. Researchers are revealing the mystery in causing of IOP with methods of genome‐wide association studies.^124^

Ferrari et al.^125^ reported that LRP5 variants are implicated in idiopathic male osteoporosis. The positivity rate of pathogenic variants is twofold higher in children compared to adults, indicating that the frequency of mutation of related genes may be related to the age of beginning. Using gene panel sequencing in diagnosis of children and young adults referred for IOP reveals that the most frequent mutation happened in LRP5, WNT1, and COL1A1 or COL1A2 genes.^126^ In the way of exploring correlated pathologic gene mutants in IOP, researchers have also found some gene mutations relevant to bone, but the mechanism still perplexing.^127^

In addition, the change in expression of bone metabolic genes also leads to IO. Patsch et al.^128^ analyzed the iliac crest biopsies of men with IO and found decreases in the expression of WNT10B, RUNX2, RANKL, and SOST. The report also found positive correlations of WNT10B with RUNX2, osteocalcin, and RANKL, which indicates potential inhibitory downstream effects on osteoblastic transcriptional activity. A set of circulating microRNAs significant have consistently regulated in patients with osteoporosis (including IOP). Among the set of microRNAs, eight miRNAs are excellent resolving devices for patients with low‐traumatic fractures, regardless of age and sex. Correlation analysis identified significant correlations between the set miRNA and P1NP (a bone turnover marker, indicating bone formation), iPTH, TRAP5b (tartrate‐resistant acid phosphatase 5b, a marker of bone resorption), osteocalcin, as well as BMD.^129^ Some of them have been demonstrated to be associated with bone microstructure and histomorphometry.^130^ However, these studies suggest that the system expression change of related genes can be the cause of IOP. With the knowledge about IOP going deep, IOP and nephrolithiasis are believed to be highly connected. Some researchers consider idiopathic nephrolithiasis and osteoporosis as two possible manifestations of a unique clinical syndrome.^131^, ^132^

Most of the pathogenic factors affect bone by affecting the differentiation, survival and function of osteoblasts and osteoclasts. In addition, these pathogenic factors interact with each other and form a complex etiologic network, which makes the treatment of osteoporosis greatly complicated.

## 6 ANTI‐OSTEOPOROSIS THERAPIES

Classic anti‐osteoporosis therapies include anti‐resorptive and anabolic agents, whose mechanisms are being clarified gradually. Based on the source, mode of action and characteristics of drugs, we divided anti‐osteoporosis therapy into three categories in this review: small molecular interventions, monoclonal antibodies, and synthetic peptides. Among them, the use of small molecule interventions has the longest history. Before the precise mechanisms underlying its action were elucidated, inorganic small molecules such as bisphosphate and strontium salts have entered the clinic. Meanwhile, more and more organic small molecular inhibitors and monoclonal antibodies based on molecular targets have been developed. Some of which have obtained sufficient clinical evidence. Besides, small molecular nutrition supplements such as vitamin D and vitamin K are recommended as effective methods to enhance BMD and reduce fracture risk. Synthetic peptides based on natural hormone structure have higher yield, stronger physiological activity, and lower immunogenicity and can be personalized modified. The available synthetic peptides, such as teriparatide and abaloparatide, have unique anabolic effects, but due to the multiple targets, their safety still needs to be further evaluated.

### 6.1 Small molecular interventions

#### 6.1.1 Bisphosphonates

**FIGURE 3:**
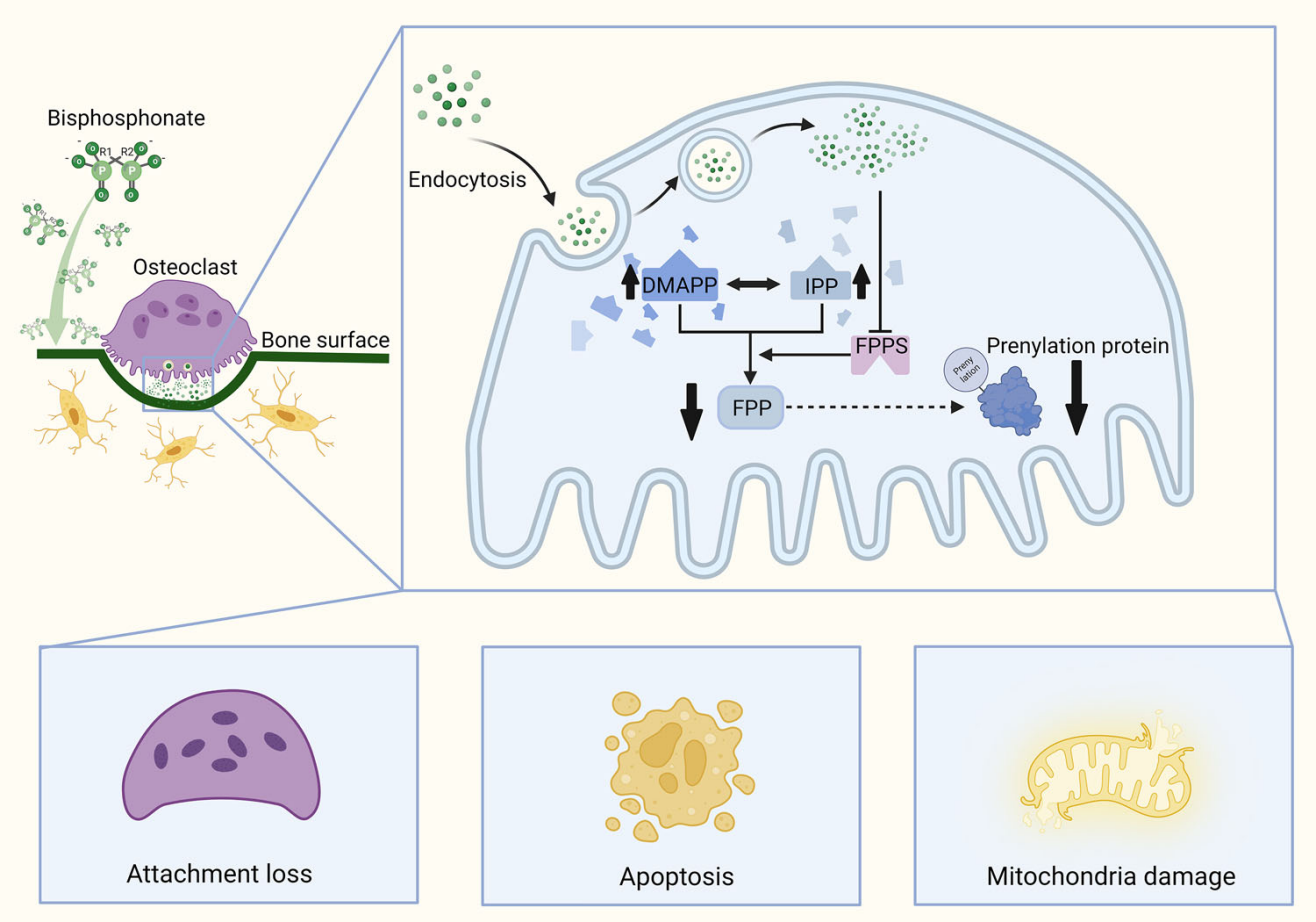
Regulation of bisphosphonates (Bps) on osteoclasts. The unique P–C–P core skeleton enables Bps to combine stably on the bone surface. After entering osteoclasts through endocytosis, Bps can inhibit farnesyl pyrophosphate synthases (FPPS) and lead to the accumulation of toxic metabolites on the upstream and the loss of downstream prenylation protein, eventually resulting in attachment loss, apoptosis, and mitochondrial damage of osteoclasts. DMAPP, dimethylallyl pyrophosphate; IPP, isopentenyl pyrophosphate.

Since it was found to inhibit bone resorption in the 1960s, bisphosphonates (Bps) have become the first‐line agents used to treat osteolytic diseases, including PMOP.^133^ Bps contain a core skeleton of P–C–P bonds and can stably complex with Ca in hydroxyapatit, leading to the ability to rapidly clear from circulation and selectively target bone^134^ (Figure 3). Bps deposited on the bone surface then act on mature osteoclasts through endocytosis during bone resorption.^135^ The side chain is the main functional area for Bps, which determines the pharmacological activity and anti‐resorptive potency. The first‐generation Bps contain simple short side chains, which can replace the β‐γ phosphoric acid group of adenosine triphosphate (ATP), producing non‐hydrolyzable ATP analog (AppCp‐type analogs of ATP) and playing a role by inhibiting ATP‐dependent intracellular enzymes.^136^ At present, nitrogen‐containing Bps with complex side chains are widely used. Nitrogen‐containing Bps can inhibit farnesyl pyrophosphate synthase in the mevalonate metabolic pathway, thus preventing the production of isoprenoid lipids, which is necessary for protein prenylation^137^ (Figure 3). The loss of isoprene will lead to protein dysfunction including small GTPase, thus affecting cell migration, adhesion, polarization, vesicular transport, and membrane ruffling, which is essential for osteoclasts^138^ (Figure 3). In addition, the accumulation of upstream metabolites and unprenylated proteins can also produce cytotoxicity, resulting in the induction of osteoclast apoptosis.

Bps can be administered orally or intravenously, which will lead to different side effects. Oral Bps has poor absorption and may cause low compliance due to gastrointestinal reactions.^5^ Giving Bps intravenously bypasses the problem of gastrointestinal intolerance, but it will multiply the risk of bisphosphonate‐related osteonecrosis of the jaw (BRONJ). A high dosage of Bp is the main cause of BRONJ. In addition, trauma and infection are closely related.^139^, ^140^ Unfortunately, statistical results show that no effective interventions for managing BRONJ have been found.^141^ Besides, using Bps for a prolonged period has been linked to an increased risk of atypical femoral fractures (AFF),^142^ which might be related to the accumulation of microdamage in bone caused by low bone turnover rate^143^ and irregular absorption in intermittent administration mode.^144^ Taking a drug holiday is an effective way to reduce the side effects after taking Bps for 3–5 years, and the dosage can be adjusted according to the examination results, including *T* score and BMD.^145^, ^146^ Although taking Bps does potentially negatively affect the remodeling of the fracture callus, it will not lead to delayed healing.^147^ So, it is still recommended to be used early in osteoporotic fractures.^148^

Currently, risedronate and ibandronate are only approved to treat PMOP. Alendronate and zoledronate are also authorized by the Food and Drug Administration (FDA) to increase bone mass in men with osteoporosis and treat glucocorticoid‐induced osteoporosis and Paget's disease of bone (a chronic osteomatoid degeneration that can cause bone expansion, deformity and strength reduction).

#### 6.1.2 Strontium salt

Strontium (Sr) is a trace element that mainly exists in skeleton system. Due to the physical and chemical similarity of Sr and Ca, Sr is probably built into the hydroxyapatite crystal to play a role. The mechanism of Sr is related to the delayed activation of calcium sensing receptors.^149^ On the one hand, Sr inhibits the maturation, TRAP expression, and hydroxyapatite resorption of osteoclasts.^150^ On the other hand, Sr promotes the differentiation and proliferation and collagen and non‐collagen protein synthesis of osteoblasts.^151^ In addition, Sr can also adjust the crosstalk between osteoblasts and osteoclasts by inhibiting the expression of RANKL.^152^ Strontium ranelate (SrR) is composed of two stable divalent strontium ions and one ranelate. Several animal experiments have shown that SrR can accelerate the healing process and improve bone mechanical properties.^153^, ^154^, ^155^ So, adding SrR into the tissue engineering scaffold to repair bone defects and promote bone regeneration under osteoporotic conditions have become a potential topical application. Including SrR‐incorporated bioceramic scaffolds,^156^ composite gelatin coatings containing SrR‐carrying halloysite nanotubes.^157^

Some randomized controlled trials (RCTs) suggest that the level of bone formation markers increased and bone destruction markers decreased in people taking SR for at least 1 year.^158^ However, oral SrR may increase the risk of thromboembolic disease,^159^ and SrR was withdrawn in 2020. At present, only strontium succinate is used to treat osteoporosis in Europe, and strontium chloride is indicated to relieve bone pain in patients with painful skeletal metastases.

#### 6.1.3 Calcitriol

Calcitriol is the activated form of vitamin D through hepatic metabolism. Together with PTH, they jointly maintain calcium and phosphorus homeostasis.^160^ Calcitriol can upregulate calcium transient receptor potential vanilloid channel 6 and calbindin‐D9k in the enterocyte, thus stimulating calcium absorption.^161^ Adequate calcium intake is considered to maintain an appropriate blood calcium concentration and prevent the mobilization of bone calcium, thus reducing bone resorption and slowing bone loss.^162^ High extracellular calcium can induce osteoblast proliferation^163^ and inhibit osteoclasts by regulating RANKL.^164^, ^165^ Besides, vitamin D can also directly inhibit osteoclast differentiation, fusion, and bone resorption in a dose‐dependent manner.^166^

Although the beneficial effect of vitamin D on bone has been confirmed in animals,^167^ there is no clinical evidence that substantially higher doses of vitamin D confer any advantage. On the contrary, calcium or vitamin D supplements alone may cause the risk of cardiovascular^168^ and renal stones.^169^ In an ancillary study of VITAL study, the researchers suggested that taking vitamin D3 alone did not reduce fracture risk in generally healthy midlife and older adults.^170^ Similar findings are confirmed in community adults without a known history of vitamin D deficiency, osteoporosis, or fracture.^169^ The effect of combined use of vitamin D and calcium to prevent fracture remains to be verified.^171^

In Europe, a compound tablet of cholecalciferol and calcium carbonate is used to prevent skeletal‐related events in patients with advanced malignancies and the combination treatment of osteoporosis with alendronate, cholecalciferol, and ibandronate. Currently, 400–800 units of vitamin D and 1000–1200 mg of calcium are recommended to be taken together daily, including the total amount obtained from food and supplements.^172^, ^173^

#### 6.1.4 Menaquinones

Vitamin K was first found to be associated with bone metabolism in warfarin anticoagulant patients. Menaquinones or vitamin K2 is a carboxylated coenzyme of several vitamin K‐dependent proteins, including osteocalcin.^174^ Carboxylated osteocalcin can induce calcium deposition on the surface of hydroxyapatite, thus enhancing bone mineralization.^175^ While a high level of undercarboxylated osteocalcin in serum is thought to be a risk of osteoporotic fracture in elderly women.^176^ A meta‐analysis indicated that menaquinone supplementation can maintain BMD and reduce fracture rate in postmenopausal women, which can be an option to counter bone loss.^175^, ^177^ However, there is no guideline for the recommended doses and forms of vitamin K in the prevention of osteoporosis. Some scholars believe that 155–188 μg vitamin K should be consumed each day to maintain bone metabolism, while others believe that the carboxylation of osteocalcin requires at least 250 μg vitamin K intake.^178^ Since no toxicity data are available, the maximum vitamin K intake is not clearly defined.^179^

At present, menaquinones are mainly used to treat coagulation dysfunction and are only approved for the treatment of osteoporosis in several Asian countries, including Japan and China. Vitamin K supplementation from plants and dairy products should be considered as a preventive measure.^180^

#### 6.1.5 SERMs

Earlier in the article, we discussed that estrogen deficiency is the leading cause of osteoporosis in postmenopausal women. Estrogen is an important protective factor that can simultaneously regulate osteoblasts and osteoclasts and participate in their crosstalk. Hormone‐related therapies using estrogen and progesterone have been shown to reduce estrogen deficiency‐mediated increased bone turnover and prevent further bone loss.^181^ However, due to risks such as breast cancer exceeding benefits, hormonal replacement therapy is only recommended for the relief of menopausal symptoms in the lowest dose necessary and for the shortest time possible,^181^ and estrogen has been replaced by SERMs. SERMs can produce different conformational changes through structural changes and binding with different ERs in different tissues. SERMs act as estrogenic agonists in bone and can antagonize estrogen in breast and uterus, which can effectively reduce estrogen‐related risks (Figure 2).

To improve the bioavailability and reduce the dosage, a variety of new formulations and administrations have been reported. Examples include an oral raloxifene‐loaded bioadhesive nanoparticle made up of Carbopol 940, glyceryl distearate, and D‐α‐tocopheryl polyethylene glycol 1000 succinate (TGPS),^182^ an intranasally gelation raloxifene‐loaded chitosan nanoparticles,^183^ a raloxifene‐loaded nasal delivery misemgel matrix using nanosized self‐emulsifying systems,^184^ an intravenous human serum albumin‐based nanoparticles loaded with raloxifene,^185^ a menthol added transdermal formulation containing raloxifene nanoparticles,^186^ all of which have yielded demonstrated results in vivo. In addition, some plant‐based estrogen‐like substances extracted from herbs show similar functions with SERMs. Herba epimedii and its extracted icariin have been widely reported to be able to activate non‐genomic ERα signaling selectively and simultaneously affect osteoblasts and BMSCs.^187^, ^188^ Wang et al.^189^ found a natural product, norlichexanthone, which is an ERα ligand and can exert the therapeutic effect with less estrogen activity.

Currently approved SERMs include tamoxifen, toremifene, raloxifene, ospemifene, bazedoxifene, and lasofoxifene. Tamoxifen and toremifene are first used to treat ER^+^ breast cancer and prevent the breast cancer in high‐risk adult women. Both of them are not approved for the treatment of osteoporosis due to their ability to activate endometrial ERs and increase the endometrial cancer risk.^190^ Being a partial agonist in the endometrium, ospemifene has positive effects on vaginal dryness and can be used to treat vulvovaginal atrophy.^191^ Raloxifene is most widely used in the prevention and treatment of PMOP and has been shown to significantly reduce breaks in the spine but not in the hip fractures in PMOP. Bazedoxifene, together with conjugated estrogens, is used in PMOP at risk of fracture. In Europe, lasofoxifene is approved as a third‐generation SERM for osteoporosis with increased fracture risk, but its efficacy and adverse reactions still need further evaluation.^192^

#### 6.1.6 RANKL inhibitors

**FIGURE 4:**
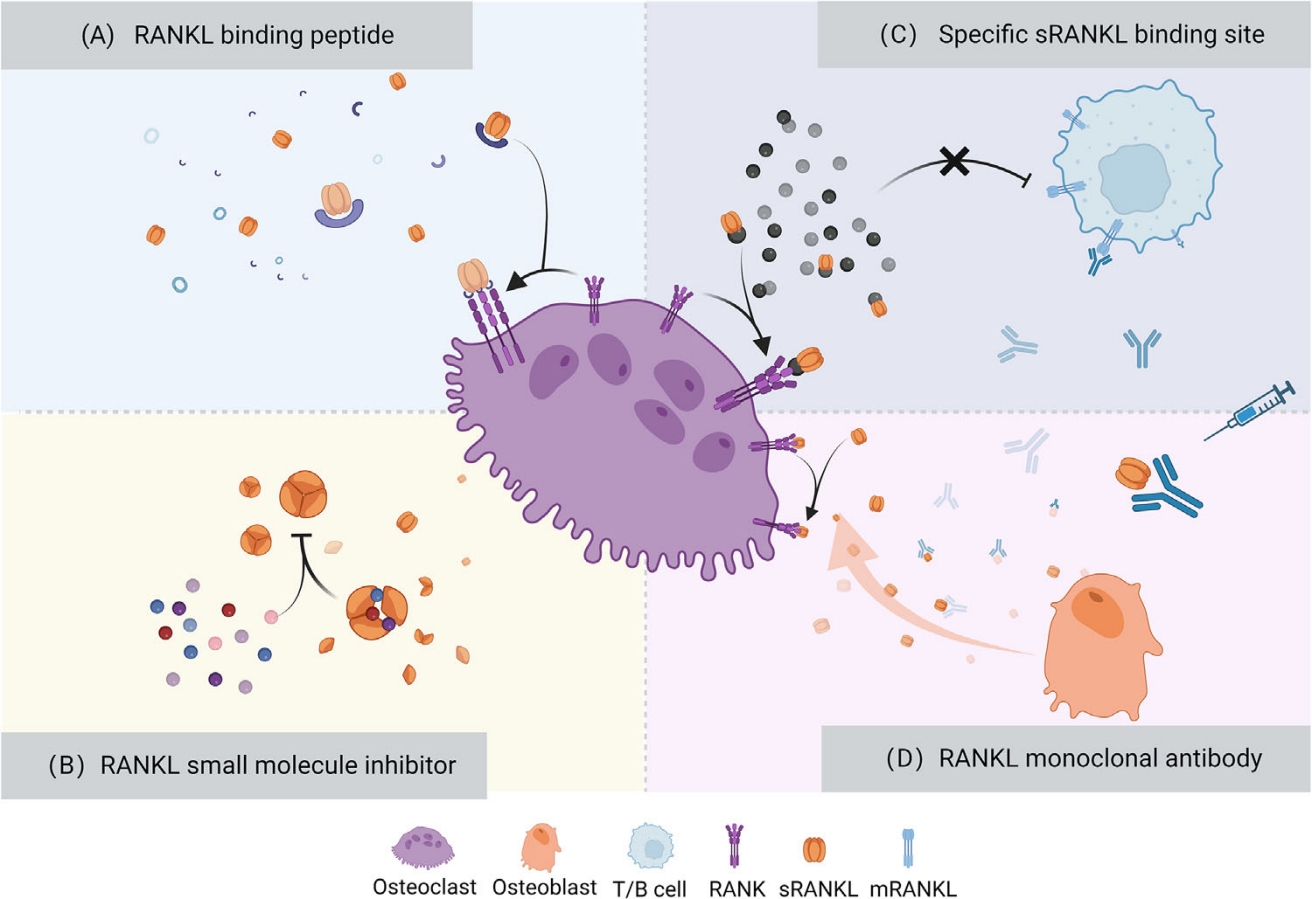
Action mode of pharmaceuticals targeting receptor activator of nuclear kappa‐B ligand (RANKL). (A) RANKL‐binding peptide has a structure similar to osteoprotegerin (OPG), which can induce topological changes in the RANK structure after binding with RANKL. (B) RANKL small molecule inhibitors can prevent the formation of RANKL trimer. (C) A newly discovered small molecule S3‐15 can specifically target soluble RANKL and block its binding with RANK without affecting membrane RANKL expressed on T or B cells. (D) The injected RANKL monoclonal antibodies neutralize RANK.

The imbalance of RANKL and osteoprotegerin (OPG) is an essential manifestation of bone homeostasis destruction. Many studies have proved that targeted RANKL therapy is an effective way to treat osteolytic diseases. In this review, we discuss different methods to inhibit RANKL and RANK binding (Figure 4).

Miyata et al.^193^ reported a pyridinylpyrimidine derivative (AS2676293) through high‐throughput screening that can rescue rapid bone loss in RANKL‐injected mice. This team further developed AS2690168 with a similar structure. In ovariectomized (OVX) mice, AS2690168 can inhibit the decrease in BMD in a dose‐dependent manner without affecting the serum osteocalcin level.^194^ In addition to osteoporosis, AS2676293 also shows a good therapeutic effect in rats with osteolytic diseases such as fibrous dysplasia^195^ and bone metastasis.^196^

Melagraki et al.^197^ constructed a computer virtual screening system based on structural modeling using known TNF inhibitors. Using this system, Melagraki's team found that T8 and T23 with the function of directly inhibiting TNF and RANKL at the same time and conducted molecular dynamics calculations and in vitro evaluations. After that, they identified another plant‐origin small molecule inhibitor, A11.^198^ With a large surface area and extended hydrophobic region, A11 shows typical characteristics of effective inhibitors of protein–protein interaction.^198^

Jiang et al.^199^ synthesized a porphyrin derivative, Y1599. Compared with T8 and T32, Y1599 has higher selectivity for RANKL and can inhibit RANKL‐induced bone resorption by downregulating the c‐fos/NFATc1 signaling pathway and osteoclast marker genes. Compound Y1599 is further cyclized and oxidized to obtain Y1693. Orally, Y1693 demonstrates good tolerability and efficacy in OVX mice and could also suppress the expression of osteoclast marker genes.^200^ In addition, another verteporfin analog shows a dose‐dependent inhibition of RANK–RANKL interaction in a competitive ELISA.^201^ However, its specificity has not been tested, and there is a lack of in vivo data.^201^

SPD304 was first reported to promote the dissociation of RANKL trimer (Figure 4B), but it is interrupted due to the high toxicity.^202^ Rinotas et al.^203^ developed several analogs of SPD‐304 with improved toxicity profiles, which can selectively inhibit RANKL‐induced osteoclastogenesis, without affecting TNF activity or osteoblast differentiation.

In a recent study, Huang et al.^204^ identified a binding site on soluble RANKL instead of membrane RANKL and discovered a series of soluble RANKL inhibitors (Figure 4C). Because T and B cells express membrane RANKL at the same time,^205^ targeting soluble RANKL can reduce the side effects of immunity, showing the potential to be superior to antibodies.

#### 6.1.7 Sclerostin inhibitors

At present, several potential sclerostin inhibitors screened by computer virtual screening have been reported, but the functional validation of animal and cell experiments has not been conducted. It includes a small molecular cluster^206^ and a group of herbal compounds with an aromatic group,^207^ targeting the loop 2 domain in sclerostin. A quinoxaline derivative targeting LRP5/6–sclerotin interaction.^208^

### 6.2 Monoclonal antibodies

#### 6.2.1 Anti‐RANKL antibodies

Denosumab is the first approved human IgG2 monoclonal antibody against RANKL for treating osteoporosis with high fracture risk (Figure 4D). A 60 mg dose is administered subcutaneously every 6 months. In the past decade, denosumab has consistently improved BMD and maintained a low rate of fracture risk.^209^ However, the effect of denosumab on bone homeostasis is reversible. The bone turnover rate would rapidly rebounds after discontinuation and whether the rebound is proportional to duration is not yet clear.^210^

In addition to the common side effects of biological therapy, denosumab also produces rare ONJ and AFF similar to Bps. A 7‐year FREEDOM extension indicates that the benefit–risk ratio is 281 for AFF and 40 for ONJ.^211^ A retrospective cohort study shows that denosumab was the most common second course of treatment prescribed among patients placed on a drug holiday due to taking Bps first.^212^ Conversely, taking SERMs after denosumab is still controversial, and Bps are more recommended to recover BMD loss.^213^, ^214^

At present, anti‐RANKL antibodies used to treat osteoporosis in phase III clinical trials on ClinicalTrails.gov include MW031 (NCT05215977), LY06006 (NCT05060406), QL1206 (NCT04128163), and CMAB807 (NCT03925051). Besides, some novel modified RANKL variants are reported to induce anti‐RANKL immune response as an immunogen, suggesting that immunotherapy can be used to treat osteoporosis.^215^, ^216^

#### 6.2.2 Anti‐sclerostin antibodies

Romosozumab is the only approved human monoclonal sclerostin antibody and it is used to treat high‐risk PMOP patients. Other anti‐sclerostin monoclonal antibodies under research include blosozumab (already finished phase II clinical trial),^217^ and SHR‐1222,^218^ a humanized IgG4 monoclonal antibody in a phase I clinical trial. In addition, setrusumab was not developed for osteoporosis when entering the phase III clinical trial.^219^

Romosozumab can competitively inhibit the binding of sclerotin to LPR5/6 receptor and prevent proteasomal degradation of β‐catenin, thereby ultimately fostering the osteogenic differentiation of MSCs and inhibiting osteoclasts activity to regulate bone mass.^220^

However, a few phase III clinical trials indicated that inhibition of sclerostin may elevate cardiovascular risk, which might be related to the cardiovascular protection of sclerotin.^221^, ^222^ Meanwhile, the initial anabolic effect of anti‐sclerostin treatment may be short‐lived and the impact of romosozumab on BMD decreases after continuous use for more than 12 months.^223^ This may be because sclerostin inhibition induced an increase in endogenous Wnt antagonist Dickkopf‐1 as negative feedback.^224^ A bispecific antibody targeting sclerostin and Dickkopf‐1 may become a potential therapeutic strategy.^225^

### 6.3 Synthetic peptides

#### 6.3.1 Calcitonin

Calcitonin is a natural 32‐amino‐acid‐containing polypeptide secreted by parafollicular cells of the thyroid gland. Calcitonin controls blood calcium mainly in two ways: inhibiting the differentiation and proliferation of osteoclasts through binding to the specific receptor, and inhibiting the reabsorption of calcium and phosphorus in renal tubules.^226^ Recent studies in genetically modified mice suggest that calcitonin appears to inhibit osteoblast activity.^226^ Unlike other anti‐resorptive agents, calcitonin holds a unique advantage in analgesia and thus could be used to relieve the pain caused by osteoporosis‐related vertebral compression fractures. This function might be related to altering Na^+^ channel and serotonin receptor expression or endorphin‐mediated mechanisms, but its specific mechanism is not yet clear.^227^

The commercialized preparations of calcitonin include recombinant salmon calcitonin and elcatonin, administered intranasally, subcutaneously, or intramuscularly. Traditional calcitonin formulations may cause a sudden drop in blood calcium due to the burst release and require frequent administration because of the short half‐life and wide distribution of receptors around the whole body.^228^ Li's team continues to report a thermosensitive hydrogel with oxidized calcium alginate and hydroxypropyl chitin added as a versatile platform for sustained release of calcitonin.^229^ In this system, calcitonin can be stably released for more than 1 month after a single subcutaneous injection and shows sustained effects in bone trabecula reconstruction in glucocorticoid‐induced osteoporosis rats.^230^, ^231^ In addition, they also report a bone‐seeking hexapeptide‐conjugated salmon calcitonin whose femur tissue accumulation is threefold higher in ovariectomized models.^232^ Similarly, Wang et al.^233^ designed a polylactic acid microsphere coated with tannic acid/PEGylated‐salcatonin layer‐by‐layer films with unique zero‐order release kinetics.

A number of clinical studies suggests that there is a potential risk of malignancy associated with calcitonin use, such as basal cell carcinomas, prostate cancer, and liver cancer.^234^ Therefore, calcitonin gets the FDA approval only for the treatment of osteoporotic women at least 5 years postmenopausal without alternative treatments.^235^ In Europe, calcitonin is only used for a short term in bone metabolic disorders, including Paget's disease, acute bone loss due to sudden immobilization, and cancer‐induced hypercalcemia.^236^ Meanwhile, its oral formation (SMC021) was also terminated in the third phase clinical trial (NCT00525798).^237^

#### 6.3.2 PTH

**FIGURE 5:**
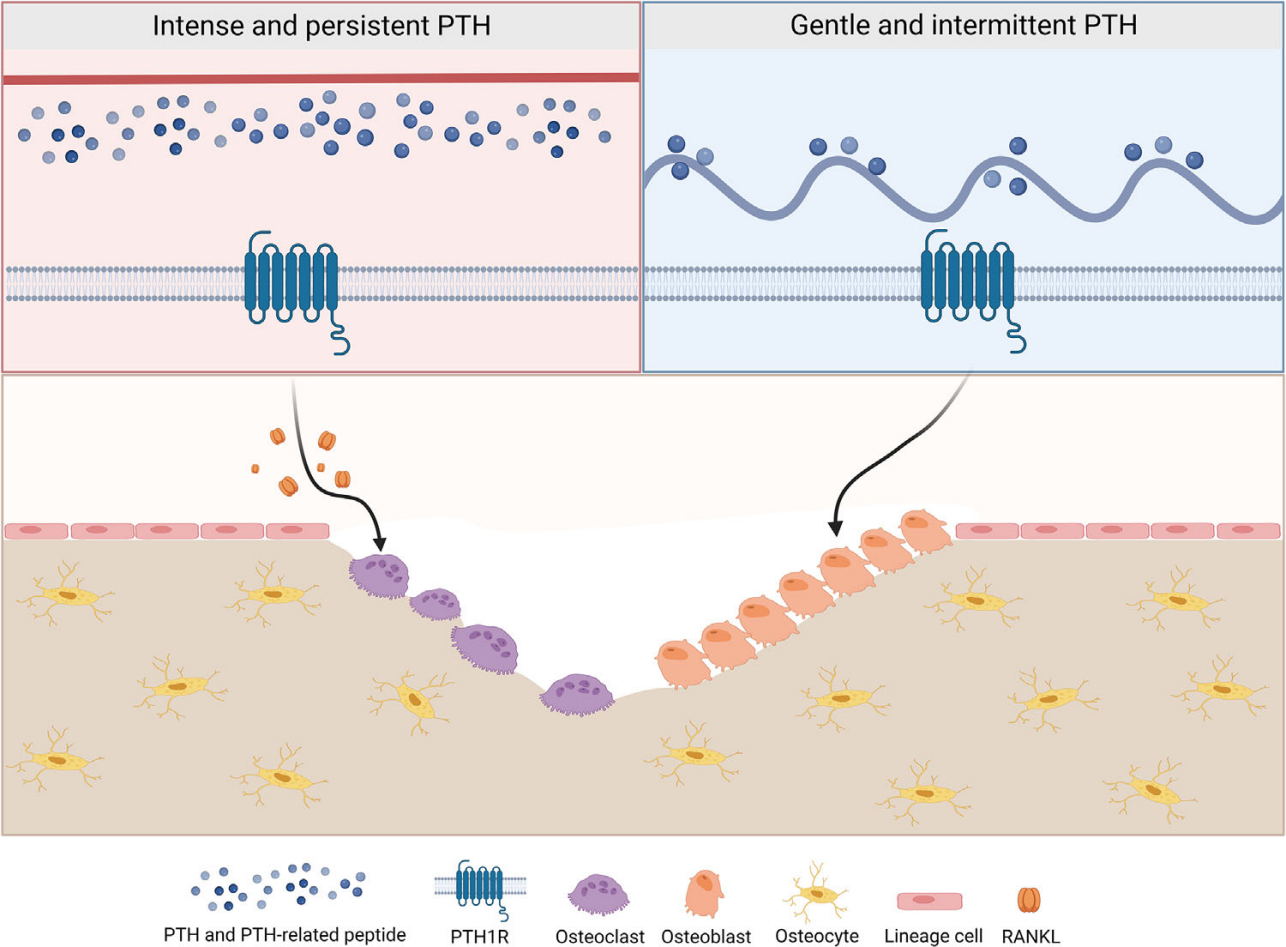
Parathyroid hormone (PTH) bidirectionally modulates bone metabolism. PTH and PTH‐related peptides play a role by combining with PTH1R in the skeleton system. Different means of administration will lead to different effects on bone tissue cells, thus producing osteoclastic or osteogenic effects. RANKL, receptor activator of nuclear kappa‐B ligand.

PTH is an 84‐amino‐acid‐containing single‐chain polypeptide secreted by the main cells of the parathyroid gland. PTH plays a vital role in calcium and phosphorus metabolism and has complex mechanisms on bone metabolism^238^ (Figure 5). In intermittent administration mode, PTH induces the osteogenic differentiation of MSCs and promotes the proliferation and differentiation of osteoblastic lineages.^239^ During continuous high‐dose administration, PTH indirectly regulates osteoclasts by mediating through RANKL secretion from osteoblasts.^240^ At present, the synthetic PTH includes teriparatide and abaloparatide, consisting of the first 34 amino acids of PTH and PTH‐related peptide (PTHrP), respectively. Unlike teriparatide, abaloparatide binds more tightly to PTH1R RG conformations, leading to a shorter cyclic adenosine monophosphate (cAMP) response.^241^ Therefore, abaloparatide causes less bone resorption than teriparatide.^241^ Recent studies in rats have also shown that abaloparatide can promote bone formation without increasing bone resorption.^242^ In addition, abaloparatide also shows greater osteogenic effects and better tolerance.^243^

Both PTH and PTHrP could cause osteosarcoma in a dose‐dependent effect when administered in high doses for 18–24 months in rats.^244^ However, a 15‐year postmarketing surveillance study shows that teriparatide does not increase adult osteosarcoma, advising to remove the black box warning regarding the potential risk of osteosarcoma.^245^, ^246^ This may be because the 2‐year treatment runs through the whole life of rats while only occupying a small part of human life. Furthermore, daily subcutaneous injection administration may be one of the reasons for poor patient compliance. Therefore, a series of experiments on oral delivery of PTH or long‐acting and slow‐released preparation is being carried out. Including a weekly dose teriparatide encapsulated dissolving microneedle patch,^247^ an orally available enteric‐microencapsulated teriparatide‐deoxycholic acid nanocomplex,^248^ a triple padlock nanocarrier prepared by a taurocholic acid‐conjugated chondroitin sulfate,^249^ and an oleic acid‐based dispersion in combination with chitosan‐teriparatide polyelectrolyte complex,^250^ whose efficacy needs further verification.

Abaloparatide is a second‐generation osteoanabolic drug that was only approved in the USA in 2017. For now, teriparatide and abaloparatide are used to treat osteoporosis at high risk for fracture or failure or intolerance to other available osteoporosis therapies, and they are not recommended to be used for more than 2 years.^251^, ^252^

#### 6.3.3 RANKL‐binding peptide

OP3‐4 is an OPG‐like peptidomimetic that shows promising effects in reducing bone loss in OVX mice.^253^ In the mouse tooth extraction model, local application of OP3‐4 can decrease the number of osteoclasts and induce new bone formation.^254^ At the same time, OP3‐4 is injected under the jaw periosteum of normal mice, and the new bone mineralization proceeds from outside to inside, indicating that OP3‐4 may participate in the early steps of accelerating osteogenesis.^255^

WP9QY is structurally similar to the cysteine‐rich domain of type I TNF receptor. It blocks the downstream signal transduction of RANK by inducing the conformational change of the RANK extracellular domain^256^ (Figure 4A). Data from in vitro experiments indicate that WP9QY can enhance the osteogenic differentiation of MSCs, stimulate the proliferation of osteoblasts and promote the apoptosis of osteoclasts.^257^, ^258^, ^259^ In OPG‐/‐mice, WP9QY suppresses osteoclastogenesis by inhibiting RANKL and enhances osteoblastogenesis by attenuating sclerostin expression in the alveolar bone.^260^

The cyclic peptide L3‐3 strongly binds to Loop3 of ectodomain RANKL and blocks RANKL‐induced osteoclast differentiation more efficiently than OP3‐4.^261^ L3‐3B further reduces the length of the peptide chain based on L3‐3. In vitro experiments show that L3‐3B can interfere with the phosphorylation of p38 and AKT in bone marrow‐derived macrophages induced by RANKL.^262^ Unfortunately, there is no experiment using L3‐3 in vivo.

### 6.4 Alternative options

At present, the pharmacological options for osteoporosis are mainly achieved by inhibiting bone absorption or promoting bone formation. However, long‐term or high‐frequency use of these drugs may bring some side effects and adverse reactions. Therefore, further researches focus on the evaluation of the dose and route of administration and combination medication. Meanwhile, progress in studies about the molecular mechanism of maintaining bone homeostasis offers opportunities for new medications, and more effective therapies on specific targets remain to be further developed. On the other hand, biological therapy via stem cells and their secretome has also received broad attention. In the following part, we will summarize the new therapeutic ideas, hoping to give some alternative options.

#### 6.4.1 Polypharmacy in osteoporosis treatment

Although the strength of the evidence may vary, almost all the drugs have been demonstrated for reducing vertebral and nonvertebral fracture risk.^24^ The selection of osteoporosis treatment should be individualized and consider a variety of factors.^263^ Among them, the combination of drugs with different mechanisms may bring additional benefits.^264^ Furthermore, the sequential use of drugs can avoid the increased risk of specific side effects and minimize the resistance caused by the long‐term use of a single drug.^265^ However, it is not recommended to use two anti‐absorbent agents at the same time because of the excessive inhibition of bone turnover and increased risk of fracture.^266^

Anabolic therapy first, followed by potent antiresorptive therapy is an ideal plan.^223^, ^267^ The benefit–risk profile of Bps or denosumab is likely to be favorable for up to 10 years in individuals at high fracture risk. Anabolic therapy for up to 12–24 months should be considered for those at high or imminent fracture risk, followed by an antiresorptive drug.^268^ But owing to economic and compliance reasons, the majority of anabolic therapies are based on dissatisfaction with previous anti‐absorption therapies, most of which had taken Bps for several years.^269^ Unfortunately, both Bps and denosumab will show progressive or transient bone loss when switching to teriparatide.^267^, ^270^ An overlap of 6–12 months of PTH is recommended instead of stopping Bps immediately when switching from Bps to prevent the transient loss of BMD in cortical sites.^271^

The combination of PTH and SERMs or denosumab appears to show continuous beneficial profits.^272^, ^273^ But it is worth noting that the combination of Bps and PTH would not bring additional benefit to BMD. In contrast, Bps may reduce the anabolic effects of PTH.^274^

Recently, Jörg et al.^275^ developed a digital framework that can predict the effects of drugs used to treat PMOP. This framework unifies the fundamental mechanisms of Bps, PTH, RANKL inhibitors, and sclerostin inhibitors. This model suggests that medication schemes eliciting a rapid BMD increase may not continuously improve BMD, cautioning that long‐term indicators such as fracture should be taken as the measurement standard instead of BMD only.^275^

#### 6.4.2 Stem cell‐based therapy

Due to the fear of rare side effects and concerns regarding long‐term efficacy, the existing anabolic agents are not as well applied. Stem cell‐based regenerative therapy is considered as a new approach to regenerating bone tissue. Among them, MSCs have attracted much attention due to their lower immunogenicity, higher proliferation, differentiation potential, and extensive access.^276^ In addition to immunomodulation and differentiation potential, MSCs also have a strong paracrine effect.^277^ Therefore, EVs containing paracrine factors, which are isolated from MSC conditioned medium, offer a strategy for cell‐free MSC therapy.^278^, ^279^

Several studies have shown that human‐derived MSCs can enhance osteogenesis and prevent OVX‐mediated bone loss in mice,^280^, ^281^, ^282^ but the specific mechanism is unclear. Wang et al.^283^ analyzed the metabolomics of mice that received BMSC transplantation, suggesting that exogenous BMSC may play an anti‐osteoporosis role by maintaining estrogen levels to mediate adipogenesis and osteoblastic metabolism in bone. Through femoral artery ligation, Wang et al.^284^ found that BMSC transplantation can promote the establishment of collateral circulation and intraosseous microcirculation at the ischemic sites, thereby inducing osteoporosis formation in OVX mice. Mei et al.^285^ proposed a novel method to screen MSCs with high migration capacity, which enhances PDGFR/Wnt/β‐catenin activity, forms more bone nodules, and partly rescues the bone loss of OVX rats.

So far, stem cell‐based therapy has mainly concentrated on animal experiments because of the uncertainty regarding the post‐transplantation fate of stem cells and their safety in recipients.^284^ Here, we summarize several clinical trials using stem cells to treat osteoporosis, none of which has entered phase III clinical trials (Table 2).

**TABLE 2:** Ongoing clinical trials of stem cell‐based therapy for osteoporosis registered in Clinical Trials.gov.

Identifier	Recruitment status	Phase	Condition or disease	Intervention/treatment	Related outcome measures	Last update	Institution
NCT05152381	Recruiting	Phase I	Osteoporosis	Cultured allogeneic adult umbilical cord‐derived MSC	Adverse events	January 5, 2022	Medical Surgical Associates Center, Antigua and Barbuda
NCT02566655	Completed	Phase I	Osteoporosis and spinal fractures	Fucosylated BMSC	Infection and neoplasia, number of new fractures, bone metabolism index, BMD and TBD, bone structure	October 8, 2020	Hospital Clínico Virgen de la Arrixaca, Spain
NCT05284604	Not yet recruiting	Phase I, Phase II	Frailty (include senile osteoporosis)	BMSC	Adherence, adverse events, BMD of lumbar spine and hip, TBD, bone strength, VAS, bone microarchitecture	September 10, 2022	Michael E. DeBakey VA Medical Center, USA
NCT05520125	Not yet recruiting	Phase I, Phase II	Segmental fracture and bone loss	MSC enriched by extracellular vesicles	Adverse events, Percent of completely recovered patients	August 29, 2022	Institute of Biophysics and Cell Engineering of National Academy of Sciences of Belarus, Belarus
NCT04501354	Unknown	Phase II	Osteoporosis	Allogeneic umbilical cord‐derived MSC	BMD, VAS	August 7, 2020	Cipto Mangunkusumo Hospital, Indonesia
NCT05018637	Enrolling by invitation	Phase II	Osteoporotic vertebral compression fracture	Wharton's jelly‐derived MSC and teriparatide	BMD, VAS, *T* score of lumbar spine and femoral neck	August 24, 2021	CHA Bundang Medical Center, Korea
NCT01532076	Terminated (slow recruitment)	Phase II	Osteoporotic fracture	Adipose tissue‐derived MSC	Adverse events, BMD, histology, dose–response	September 17, 2014	University Hospital Basel, Switzerland

Abbreviations: BMD, bone mineral density; BMSC; bone marrow mesenchymal stem cell; MSC; mesenchymal stem cell; TBD, trabecular bone density; VAS, visual analog scale.

## 7 DISCUSSION

Numerous efforts have been made to deal with osteoporosis. Nevertheless, the etiologies of osteoporosis involve a series of complex mechanisms, including osteoblasts, osteoclasts, cytokines, mechanical levers, and stem cells. In this review, we narratively demonstrate the etiologies and underlying mechanisms of osteoporosis to provide readers with inspiration about new therapies. An example of a theory–medicine transformation is denosumab, and it is believed that the discovery of denosumab is a milestone in osteoporosis treatment. The approach to developing novel therapeutics shifted from observational or accidental findings to mechanism‐based findings.^286^ Besides, we also systematically review drugs in osteoporosis treatment, including therapies and concepts, which are newly developed. We believe that these works will benefit clinical workers or pharmaceutical workers in the future.

Through our collection, we realize that there are still enigmas in the pathogenesis of osteoporosis and that drugs based on current theoretical mechanisms may not be beneficial. One of the examples is the discovery of cathepsin K, which is the function substance of osteoclast resorption.^287^ The cathepsin K inhibitor odanacatib inhibits bone resorption significantly and has once been considered a promising treatment for osteoporosis. But a multicentral, randomized, double‐blind, placebo‐controlled trial revealed an increased risk of cardiovascular events in the odanacatib group, resulting in its development discontinuing.^288^ The mechanisms related to its adverse events remain incomprehensive.

In clinical applications, individualized medication regimens should be taken into account. Osteoporosis is more likely to occur in older age groups with multiple comorbidities. Fracture risk rises in older adults with high levels of high‐density lipoprotein cholestrol.^289^ Clinical studies have shown that in obese people, the response to vitamin D is blunter.^290^ In summary, more attention should be paid in groups with systematic disease and individual therapy should be designed. Unfortunately, older adults with multiple comorbidities were typically excluded from the pivotal osteoporosis RCTs, which led to insufficient knowledge about drug therapy in patients with systemic disease.

Furthermore, it may be another way to develop by shifting the focus from using drugs to inhibit bone resorption to promoting bone synthesis or combining therapies. Studies have shown that anabolic agents have greater anti‐fracture efficacy and produce larger increases in bone density than anti‐resorptive drugs, but anabolic drugs have a shorter effective time, so the transition to anti‐resorptive drugs is required.^291^ Further investigations are needed to fully understand the mechanisms and cross‐talks of osteoporosis, in order to develop new strategies or moderate existing strategies to counteract osteoporosis and enhance the quality of life of patients.

Generally speaking, advances in the mechanisms of osteoporosis have shed light on the limitations and crises of current treatments. The uncertain and controversial insights in mechanism make the development of new approaches with high efficiency and few side effects remind challengeable. In addition, we also need to go beyond the limitations of mainstream therapies and use more personalized therapies according to the patient's etiology, lifestyle, and complications.

## AUTHOR CONTRIBUTIONS

F.Y. conceived the study. H.W., Y.C.L., F.L., and H.S.W. performed literature searching and summary. H.W., Y.C.L., F.L., and F.Y. wrote the manuscript. F.Y., F.L., and L.Y. edited the manuscript. All authors have read and approved the final manuscript.

## CONFLICT OF INTEREST STATEMENT

The authors declare no conflicts of interest.

## ETHICS STATEMENT

Ethics approval and consent to participate not applicable.

## Data Availability

All data needed to evaluate the conclusions in the paper are present in the paper.

## References

[1] Sözen T , Özışık L , Başaran NÇ. An overview and management of osteoporosis. Eur J Rheumatol. 2017;4(1):46‐56. 28293453 10.5152/eurjrheum.2016.048 PMC5335887

[2] Wright NC , Looker AC , Saag KG , et al. The recent prevalence of osteoporosis and low bone mass in the United States based on bone mineral density at the femoral neck or lumbar spine. J Bone Miner Res. 2014;29(11):2520‐2526. 24771492 10.1002/jbmr.2269 PMC4757905

[3] Zhu X , Zheng H . Factors influencing peak bone mass gain. Front Med. 2021;15(1):53‐69. 32519297 10.1007/s11684-020-0748-y

[4] Genant HK , Cooper C , Poor G , et al. Interim report and recommendations of the world health organization task‐force for osteoporosis. Osteoporos Int. 1999;10(4):259‐264. 10692972 10.1007/s001980050224

[5] Blanch J , Casado E , González J , et al. Percepción de los profesionales médicos respecto la adherencia terapéutica de los pacientes con osteoporosis. Rev Osteoporos Metab Miner. 2016;8:15‐23.

[6] Hernandez‐de Sosa N , Athanasiadis G , Malouf J , et al. Heritability of bone mineral density in a multivariate family‐based study. Calcif Tissue Int. 2014;94(6):590‐596. 24687525 10.1007/s00223-014-9852-9

[7] Mitchell BD , Yerges‐Armstrong LM . The genetics of bone loss: challenges and prospects. J Clin Endocrinol Metab. 2011;96(5):1258‐1268. 21346070 10.1210/jc.2010-2865 PMC3085199

[8] Yuan J , Tickner J , Mullin BH , et al. Advanced genetic approaches in discovery and characterization of genes involved with osteoporosis in mouse and human. Front Genet. 2019;10:288. 31001327 10.3389/fgene.2019.00288 PMC6455049

[9] Lee HK , Chaboub LS , Zhu W , et al. Daam2‐PIP5K is a regulatory pathway for Wnt signaling and therapeutic target for remyelination in the CNS. Neuron. 2015;85(6):1227‐1243. 25754822 10.1016/j.neuron.2015.02.024 PMC4402944

[10] Morris JA , Kemp JP , Youlten SE , et al. An atlas of genetic influences on osteoporosis in humans and mice. Nat Genet. 2019;51(2):258‐266. 30598549 10.1038/s41588-018-0302-x PMC6358485

[11] Yang Y , Wang S , Cong H . Association between parity and bone mineral density in postmenopausal women. BMC Womens Health. 2022;22(1):87. 35321721 10.1186/s12905-022-01662-9 PMC8944100

[12] Qian Y , Wang L , Yu L , Huang W . Pregnancy‐ and lactation‐associated osteoporosis with vertebral fractures: a systematic review. BMC Musculoskelet Disord. 2021;22(1):926. 34732196 10.1186/s12891-021-04776-7 PMC8567545

[13] Finkelstein JS , Lee H , Leder BZ , et al. Gonadal steroid‐dependent effects on bone turnover and bone mineral density in men. J Clin Invest. 2016;126(3):1114‐1125. 26901812 10.1172/JCI84137 PMC4767351

[14] Sakane EN , Vieira MCC , Lazaretti‐Castro M , Maeda SS . Predictors of poor bone microarchitecture assessed by trabecular bone score in postsurgical hypoparathyroidism. J Clin Endocrinol Metab. 2019;104(12):5795‐5803. 31305931 10.1210/jc.2019-00698

[15] Formenti AM , Tecilazich F , Giubbini R , Giustina A . Risk of vertebral fractures in hypoparathyroidism. Rev Endocr Metab Disord. 2019;20(3):295‐302. 31471845 10.1007/s11154-019-09507-x

[16] Silva BC , Bilezikian JP . Skeletal abnormalities in hypoparathyroidism and in primary hyperparathyroidism. Rev Endocr Metab Disord. 2021;22(4):789‐802. 33200346 10.1007/s11154-020-09614-0

[17] Leggate J , Farish E , Fletcher CD , McIntosh W , Hart DM , Sommerville JM . Calcitonin and postmenopausal osteoporosis. Clin Endocrinol (Oxf). 1984;20(1):85‐92. 6362926 10.1111/j.1365-2265.1984.tb00062.x

[18] Matikainen N , Pekkarinen T , Ryhänen EM , Schalin‐Jäntti C . Physiology of calcium homeostasis: an overview. Endocrinol Metab Clin North Am. 2021;50(4):575‐590. 34774235 10.1016/j.ecl.2021.07.005

[19] Frara S , di Filippo L , Doga M , Loli P , Casanueva FF , Giustina A . Novel approaches to bone comorbidity in Cushing's disease: an update. Pituitary. 2022;25(5):754‐759. 35849272 10.1007/s11102-022-01252-w

[20] Kim S‐M , Ryu V , Miyashita S , et al. Thyrotropin, hyperthyroidism, and bone mass. J Clin Endocrinol Metab. 2021;106(12):e4809‐e4821. 34318885 10.1210/clinem/dgab548 PMC8864741

[21] Hunter DJ , Sambrook PN . Bone loss. Epidemiology of bone loss. Arthritis Res. 2000;2(6):441‐445. 11094456 10.1186/ar125 PMC128872

[22] Rizzoli R , Biver E , Brennan‐Speranza TC . Nutritional intake and bone health. Lancet Diabetes Endocrinol. 2021;9(9):606‐621. 34242583 10.1016/S2213-8587(21)00119-4

[23] Polzonetti V , Pucciarelli S , Vincenzetti S , Polidori P . Dietary intake of vitamin D from dairy products reduces the risk of osteoporosis. Nutrients. 2020;12(6):1743. 10.3390/nu12061743 PMC7353177 32532150

[24] Dimai HP , Fahrleitner‐Pammer A . Osteoporosis and fragility fractures: currently available pharmacological options and future directions. Best Pract Res Clin Rheumatol. 2022;36(3):101780. 36163230 10.1016/j.berh.2022.101780

[25] Wang S‐M , Han K‐D , Kim N‐Y , et al. Association of alcohol intake and fracture risk in elderly varied by affected bones: a nationwide longitudinal study. Psychiatry Investig. 2020;17(10):1013‐1020. 10.30773/pi.2020.0143 PMC7596281 33059395

[26] Godos J , Giampieri F , Chisari E , et al. Alcohol consumption, bone mineral density, and risk of osteoporotic fractures: a dose–response meta‐analysis. Int J Environ Res Public Health. 2022;19(3):1515. 10.3390/ijerph19031515 PMC8835521 35162537

[27] Petermann‐Rocha F , Ferguson LD , Gray SR , et al. Association of sarcopenia with incident osteoporosis: a prospective study of 168,682 UK biobank participants. J Cachexia Sarcopenia Muscle. 2021;12(5):1179‐1188. 34264024 10.1002/jcsm.12757 PMC8517357

[28] Heinonen A , Kannus P , Sievänen H , et al. Randomised controlled trial of effect of high‐impact exercise on selected risk factors for osteoporotic fractures. Lancet. 1996;348(9038):1343‐1347. 8918277 10.1016/S0140-6736(96)04214-6

[29] Chen L‐R , Hou P‐H , Chen K‐H . Nutritional support and physical modalities for people with osteoporosis: current opinion. Nutrients. 2019;11(12):2848. 10.3390/nu11122848 PMC6950804 31757101

[30] Ott SM . In osteoporosis or osteopenia, exercise interventions improve BMD; effects vary by exercise type and BMD site. Ann Intern Med. 2022;175(4):JC46. 35377720 10.7326/J22-0014

[31] Jones G , Nguyen T , Sambrook PN , Kelly PJ , Gilbert C , Eisman JA . Symptomatic fracture incidence in elderly men and women: the Dubbo osteoporosis epidemiology study (DOES). Osteoporos Int. 1994;4(5):277‐282. 7812076 10.1007/BF01623352

[32] Curry SJ , Krist AH , Owens DK , et al. Screening for osteoporosis to prevent fractures: US preventive services task force recommendation statement. JAMA. 2018;319(24):2521‐2531. 29946735 10.1001/jama.2018.7498

[33] Väänänen HK , Härkönen PL . Estrogen and bone metabolism. Maturitas. 1996;23(suppl):S65‐S69. 8865143 10.1016/0378-5122(96)01015-8

[34] Recker R , Lappe J , Davies KM , Heaney R . Bone remodeling increases substantially in the years after menopause and remains increased in older osteoporosis patients. J Bone Miner Res. 2004;19(10):1628‐1633. 15355557 10.1359/JBMR.040710

[35] Nordin BEC , Wishart JM , Clifton PM , et al. A longitudinal study of bone‐related biochemical changes at the menopause. Clin Endocrinol (Oxf). 2004;61(1):123‐130. 15212654 10.1111/j.1365-2265.2004.02066.x

[36] Khosla S , Melton LJ , Atkinson EJ , O'Fallon WM , Klee GG , Riggs BL . Relationship of serum sex steroid levels and bone turnover markers with bone mineral density in men and women: a key role for bioavailable estrogen. J Clin Endocrinol Metab. 1998;83(7):2266‐2274. 9661593 10.1210/jcem.83.7.4924

[37] Jensen EV , Desombre ER , Kawashima T , Suzuki T , Kyser K , Jungblut PW . Estrogen‐binding substances of target tissues. Science. 1967;158(3800):529‐530. 10.1126/science.158.3800.529-c 17749092

[38] Jensen EV , Suzuki T , Kawashima T , Stumpf WE , Jungblut PW , DeSombre ER . A two‐step mechanism for the interaction of estradiol with rat uterus. Proc Natl Acad Sci U S A. 1968;59(2):632‐638. 5238991 10.1073/pnas.59.2.632 PMC224719

[39] Nilsson S , Mäkelä S , Treuter E , et al. Mechanisms of estrogen action. Physiol Rev. 2001;81(4):1535‐1565. 11581496 10.1152/physrev.2001.81.4.1535

[40] Yakimchuk K , Jondal M , Okret S . Estrogen receptor α and β in the normal immune system and in lymphoid malignancies. Mol Cell Endocrinol. 2013;375(1‐2):121‐129. 23707618 10.1016/j.mce.2013.05.016

[41] Khosla S , Atkinson EJ , Melton LJ , Riggs BL . Effects of age and estrogen status on serum parathyroid hormone levels and biochemical markers of bone turnover in women: a population‐based study. J Clin Endocrinol Metab. 1997;82(5):1522‐1527. 9141544 10.1210/jcem.82.5.3946

[42] Pfeilschifter J , Chenu C , Bird A , Mundy GR , Roodman GD . Interleukin‐1 and tumor necrosis factor stimulate the formation of human osteoclastlike cells in vitro. J Bone Miner Res. 1989;4(1):113‐118. 2785743 10.1002/jbmr.5650040116

[43] Kwan Tat S , Padrines M , Théoleyre S , Heymann D , Fortun Y . IL‐6, RANKL, TNF‐alpha/IL‐1: interrelations in bone resorption pathophysiology. Cytokine Growth Factor Rev. 2004;15(1):49‐60. 14746813 10.1016/j.cytogfr.2003.10.005

[44] Fischer V , Haffner‐Luntzer M . Interaction between bone and immune cells: implications for postmenopausal osteoporosis. Semin Cell Dev Biol. 2022;123:14‐21. 34024716 10.1016/j.semcdb.2021.05.014

[45] Tang M , Lu L , Yu X . Interleukin‐17A interweaves the skeletal and immune systems. Front Immunol. 2020;11:625034. 33613566 10.3389/fimmu.2020.625034 PMC7890031

[46] Takeuchi T , Yoshida H , Tanaka S . Role of interleukin‐6 in bone destruction and bone repair in rheumatoid arthritis. Autoimmun Rev. 2021;20(9):102884. 34229044 10.1016/j.autrev.2021.102884

[47] Roato I , Gorassini E , Brunetti G , et al. IL‐7 modulates osteoclastogenesis in patients affected by solid tumors. Ann N Y Acad Sci. 2007;1117:377‐384. 17584976 10.1196/annals.1402.002

[48] Amarasekara DS , Yun H , Kim S , Lee N , Kim H , Rho J . Regulation of osteoclast differentiation by cytokine networks. Immune Netw. 2018;18(1):e8. 29503739 10.4110/in.2018.18.e8 PMC5833125

[49] Weitzmann MN , Roggia C , Toraldo G , Weitzmann L , Pacifici R . Increased production of IL‐7 uncouples bone formation from bone resorption during estrogen deficiency. J Clin Invest. 2002;110(11):1643‐1650. 12464669 10.1172/JCI15687 PMC151629

[50] Yao Z , Getting SJ , Locke IC . Regulation of TNF‐induced osteoclast differentiation. Cells. 2021;11(1) 10.3390/cells11010132 PMC8750957 35011694

[51] Almeida M , Laurent MR , Dubois V , et al. Estrogens and androgens in skeletal physiology and pathophysiology. Physiol Rev. 2017;97(1):135‐187. 27807202 10.1152/physrev.00033.2015 PMC5539371

[52] Shevde NK , Bendixen AC , Dienger KM , Pike JW . Estrogens suppress RANK ligand‐induced osteoclast differentiation via a stromal cell independent mechanism involving c‐Jun repression. Proc Natl Acad Sci U S A. 2000;97(14):7829‐7834. 10869427 10.1073/pnas.130200197 PMC16630

[53] Nakamura T , Imai Y , Matsumoto T , et al. Estrogen prevents bone loss via estrogen receptor alpha and induction of Fas ligand in osteoclasts. Cell. 2007;130(5):811‐823. 17803905 10.1016/j.cell.2007.07.025

[54] Gavali S , Gupta MK , Daswani B , Wani MR , Sirdeshmukh R , Khatkhatay MI . Estrogen enhances human osteoblast survival and function via promotion of autophagy. Biochim Biophys Acta Mol Cell Res. 2019;1866(9):1498‐1507. 31255720 10.1016/j.bbamcr.2019.06.014

[55] Matsumoto Y , Otsuka F , Takano‐Narazaki M , et al. Estrogen facilitates osteoblast differentiation by upregulating bone morphogenetic protein‐4 signaling. Steroids. 2013;78(5):513‐520. 23499826 10.1016/j.steroids.2013.02.011

[56] Yeh C‐R , Chiu J‐J , Lee C‐I , et al. Estrogen augments shear stress‐induced signaling and gene expression in osteoblast‐like cells via estrogen receptor‐mediated expression of beta1‐integrin. J Bone Miner Res. 2010;25(3):627‐639. 19821775 10.1359/jbmr.091008 PMC3153398

[57] Robinson LJ , Yaroslavskiy BB , Griswold RD , et al. Estrogen inhibits RANKL‐stimulated osteoclastic differentiation of human monocytes through estrogen and RANKL‐regulated interaction of estrogen receptor‐alpha with BCAR1 and Traf6. Exp Cell Res. 2009;315(7):1287‐1301. 19331827 10.1016/j.yexcr.2009.01.014 PMC2765696

[58] Gavali S , Gupta MK , Daswani B , Wani MR , Sirdeshmukh R , Khatkhatay MI . LYN, a key mediator in estrogen‐dependent suppression of osteoclast differentiation, survival, and function. Biochim Biophys Acta Mol Basis Dis. 2019;1865(3):547‐557. 30579930 10.1016/j.bbadis.2018.12.016

[59] Miyauchi Y , Sato Y , Kobayashi T , et al. HIF1α is required for osteoclast activation by estrogen deficiency in postmenopausal osteoporosis. Proc Natl Acad Sci U S A. 2013;110(41):16568‐16573. 24023068 10.1073/pnas.1308755110 PMC3799362

[60] Cooper LF , Tiffee JC , Griffin JP , Hamano H , Guo Z . Estrogen‐induced resistance to osteoblast apoptosis is associated with increased hsp27 expression. J Cell Physiol. 2000;185(3):401‐7. 11056010 10.1002/1097-4652(200012)185:3<401::AID-JCP10>3.0.CO;2-C

[61] Almeida M , Iyer S , Martin‐Millan M , et al. Estrogen receptor‐α signaling in osteoblast progenitors stimulates cortical bone accrual. J Clin Invest. 2013;123(1):394‐404. 23221342 10.1172/JCI65910 PMC3533305

[62] Schiavi J , Fodera DM , Brennan MA , McNamara LM . Estrogen depletion alters osteogenic differentiation and matrix production by osteoblasts in vitro. Exp Cell Res. 2021;408(1):112814. 34492267 10.1016/j.yexcr.2021.112814

[63] Pacifici R . Estrogen deficiency, T cells and bone loss. Cell Immunol. 2008;252(1‐2):68‐80. 17888417 10.1016/j.cellimm.2007.06.008

[64] Mansour A , Anginot A , Mancini SJC , et al. Osteoclast activity modulates B‐cell development in the bone marrow. Cell Res. 2011;21(7):1102‐1115. 21321604 10.1038/cr.2011.21 PMC3193501

[65] Grimaldi CM , Cleary J , Dagtas AS , Moussai D , Diamond B . Estrogen alters thresholds for B cell apoptosis and activation. J Clin Invest. 2002;109(12):1625‐1633. 12070310 10.1172/JCI14873 PMC151010

[66] Horowitz MC , Bothwell ALM , Hesslein DGT , Pflugh DL , Schatz DG . B cells and osteoblast and osteoclast development. Immunol Rev. 2005;208:141‐153. 16313346 10.1111/j.0105-2896.2005.00328.x

[67] Eghbali‐Fatourechi G , Khosla S , Sanyal A , Boyle WJ , Lacey DL , Riggs BL . Role of RANK ligand in mediating increased bone resorption in early postmenopausal women. J Clin Invest. 2003;111(8):1221‐1230. 12697741 10.1172/JCI17215 PMC152939

[68] Wu D , Cline‐Smith A , Shashkova E , Perla A , Katyal A , Aurora R. T‐cell mediated inflammation in postmenopausal osteoporosis. Front Immunol. 2021;12:687551. 34276675 10.3389/fimmu.2021.687551 PMC8278518

[69] Tyagi AM , Mansoori MN , Srivastava K , et al. Enhanced immunoprotective effects by anti‐IL‐17 antibody translates to improved skeletal parameters under estrogen deficiency compared with anti‐RANKL and anti‐TNF‐α antibodies. J Bone Miner Res. 2014;29(9):1981‐1992. 24677326 10.1002/jbmr.2228

[70] Kobayashi K , Takahashi N , Jimi E , et al. Tumor necrosis factor alpha stimulates osteoclast differentiation by a mechanism independent of the ODF/RANKL–RANK interaction. J Exp Med. 2000;191(2):275‐286. 10637272 10.1084/jem.191.2.275 PMC2195746

[71] Ershler WB , Harman SM , Keller ET . Immunologic aspects of osteoporosis. Dev Comp Immunol. 1997;21(6):487‐499. 9463782 10.1016/s0145-305x(97)00029-3

[72] van't Hof RJ , Ralston SH . Nitric oxide and bone. Immunology. 2001;103(3):255‐261. 11454054 10.1046/j.1365-2567.2001.01261.x PMC1783253

[73] Levescot A , Chang MH , Schnell J , et al. IL‐1β‐driven osteoclastogenic Tregs accelerate bone erosion in arthritis. J Clin Invest. 2021;131(18) 10.1172/JCI141008 PMC8439607 34343136

[74] Manolagas SC . From estrogen‐centric to aging and oxidative stress: a revised perspective of the pathogenesis of osteoporosis. Endocr Rev. 2010;31(3):266‐300. 20051526 10.1210/er.2009-0024 PMC3365845

[75] Li L , Wang Z . Ovarian aging and osteoporosis. Adv Exp Med Biol. 2018;1086:199‐215. 30232761 10.1007/978-981-13-1117-8_13

[76] Greenberg C , Kukreja SC , Bowser EN , Hargis GK , Henderson WJ , Williams GA . Parathyroid hormone secretion: effect of estradiol and progesterone. Metabolism. 1987;36(2):151‐154. 3807787 10.1016/0026-0495(87)90009-6

[77] He J , Xu S , Zhang B , et al. Gut microbiota and metabolite alterations associated with reduced bone mineral density or bone metabolic indexes in postmenopausal osteoporosis. Aging (Albany NY). 2020;12(9):8583‐8604. 32392181 10.18632/aging.103168 PMC7244073

[78] Xu L , Liu Y , Sun Y , et al. Tissue source determines the differentiation potentials of mesenchymal stem cells: a comparative study of human mesenchymal stem cells from bone marrow and adipose tissue. Stem Cell Res Ther. 2017;8(1):275. 29208029 10.1186/s13287-017-0716-x PMC5718061

[79] Li C‐J , Xiao Y , Yang M , et al. Long noncoding RNA Bmncr regulates mesenchymal stem cell fate during skeletal aging. J Clin Invest. 2018;128(12):5251‐5266. 30352426 10.1172/JCI99044 PMC6264619

[80] Turinetto V , Vitale E , Giachino C . Senescence in human mesenchymal stem cells: functional changes and implications in stem cell‐based therapy. Int J Mol Sci. 2016;17(7):1164. 10.3390/ijms17071164 PMC4964536 27447618

[81] d'Adda di Fagagna F , Reaper PM , Clay‐Farrace L , et al. A DNA damage checkpoint response in telomere‐initiated senescence. Nature. 2003;426(6963):194‐198. 14608368 10.1038/nature02118

[82] Herbig U , Jobling WA , Chen BPC , Chen DJ , Sedivy JM . Telomere shortening triggers senescence of human cells through a pathway involving ATM, p53, and p21(CIP1), but not p16(INK4a). Mol Cell. 2004;14(4):501‐513. 15149599 10.1016/s1097-2765(04)00256-4

[83] Ma Y , Qi M , An Y , et al. Autophagy controls mesenchymal stem cell properties and senescence during bone aging. Aging Cell. 2018;17(1) 10.1111/acel.12709 PMC5770781 29210174

[84] Diao Z , Ji Q , Wu Z , et al. SIRT3 consolidates heterochromatin and counteracts senescence. Nucleic Acids Res. 2021;49(8):4203‐4219. 33706382 10.1093/nar/gkab161 PMC8096253

[85] Li X , Hong Y , He H , et al. FGF21 mediates mesenchymal stem cell senescence via regulation of mitochondrial dynamics. Oxid Med Cell Longev. 2019;2019:4915149. 31178962 10.1155/2019/4915149 PMC6501200

[86] Liu H , Xia X , Li B . Mesenchymal stem cell aging: mechanisms and influences on skeletal and non‐skeletal tissues. Exp Biol Med (Maywood). 2015;240(8):1099‐1106. 26088863 10.1177/1535370215591828 PMC4935286

[87] Infante A , Rodríguez CI . Cell and cell‐free therapies to counteract human premature and physiological aging: MSCs come to light. J Pers Med. 2021;11(10):1043. 10.3390/jpm11101043 PMC8541473 34683184

[88] Kretlow JD , Jin Y‐Q , Liu W , et al. Donor age and cell passage affects differentiation potential of murine bone marrow‐derived stem cells. BMC Cell Biol. 2008;9:60. 18957087 10.1186/1471-2121-9-60 PMC2584028

[89] Rodier F , Coppé J‐P , Patil CK , et al. Persistent DNA damage signalling triggers senescence‐associated inflammatory cytokine secretion. Nat Cell Biol. 2009;11(8):973‐979. 19597488 10.1038/ncb1909 PMC2743561

[90] Li H , Liu P , Xu S , et al. FOXP1 controls mesenchymal stem cell commitment and senescence during skeletal aging. J Clin Invest. 2017;127(4):1241‐1253. 28240601 10.1172/JCI89511 PMC5373872

[91] Falconi D , Oizumi K , Aubin JE . Leukemia inhibitory factor influences the fate choice of mesenchymal progenitor cells. Stem Cells. 2007;25(2):305‐312. 17284649 10.1634/stemcells.2006-0417

[92] Beresford JN , Bennett JH , Devlin C , Leboy PS , Owen ME . Evidence for an inverse relationship between the differentiation of adipocytic and osteogenic cells in rat marrow stromal cell cultures. J Cell Sci. 1992;102(pt 2):341‐351. 1400636 10.1242/jcs.102.2.341

[93] Dorronsoro A , Santiago FE , Grassi D , et al. Mesenchymal stem cell‐derived extracellular vesicles reduce senescence and extend health span in mouse models of aging. Aging Cell. 2021;20(4):e13337. 33728821 10.1111/acel.13337 PMC8045949

[94] Lei Q , Gao F , Liu T , et al. Extracellular vesicles deposit to rejuvenate aged bone marrow‐derived mesenchymal stem cells and slow age‐related degeneration. Sci Transl Med. 2021;13(578):eaaz8697. 10.1126/scitranslmed.aaz8697 33504653

[95] Zhang S , Chuah SJ , Lai RC , Hui JHP , Lim SK , Toh WS . MSC exosomes mediate cartilage repair by enhancing proliferation, attenuating apoptosis and modulating immune reactivity. Biomaterials. 2018;156:16‐27. 29182933 10.1016/j.biomaterials.2017.11.028

[96] Zhang Y , Chen J , Fu H , et al. Exosomes derived from 3D‐cultured MSCs improve therapeutic effects in periodontitis and experimental colitis and restore the Th17 cell/Treg balance in inflamed periodontium. Int J Oral Sci. 2021;13(1):43. 34907166 10.1038/s41368-021-00150-4 PMC8671433

[97] Liu R , Li X , Zhang Z , et al. Allogeneic mesenchymal stem cells inhibited T follicular helper cell generation in rheumatoid arthritis. Sci Rep. 2015;5:12777. 26259824 10.1038/srep12777 PMC4531289

[98] Luque‐Campos N , Contreras‐López RA , Jose Paredes‐Martínez M , et al. Mesenchymal stem cells improve rheumatoid arthritis progression by controlling memory T cell response. Front Immunol. 2019;10:798. 31040848 10.3389/fimmu.2019.00798 PMC6477064

[99] Martinet L , Fleury‐Cappellesso S , Gadelorge M , et al. A regulatory cross‐talk between Vgamma9Vdelta2 T lymphocytes and mesenchymal stem cells. Eur J Immunol. 2009;39(3):752‐762. 19197941 10.1002/eji.200838812

[100] Bi Y , Lin X , Liang H , et al. Human adipose tissue‐derived mesenchymal stem cells in Parkinson's disease: inhibition of t helper 17 cell differentiation and regulation of immune balance towards a regulatory T cell phenotype. Clin Interv Aging. 2020;15:1383‐1391. 32884248 10.2147/CIA.S259762 PMC7434526

[101] Chinnadurai R , Rajan D , Ng S , et al. Immune dysfunctionality of replicative senescent mesenchymal stromal cells is corrected by IFNγ priming. Blood Adv. 2017;1(11):628‐643. 28713871 10.1182/bloodadvances.2017006205 PMC5507374

[102] Pinho S , Frenette PS . Haematopoietic stem cell activity and interactions with the niche. Nat Rev Mol Cell Biol. 2019;20(5):303‐320. 30745579 10.1038/s41580-019-0103-9 PMC6483843

[103] Tyner SD , Venkatachalam S , Choi J , et al. p53 mutant mice that display early ageing‐associated phenotypes. Nature. 2002;415(6867):45‐53. 11780111 10.1038/415045a

[104] Wang X , Kua H‐Y , Hu Y , et al. p53 functions as a negative regulator of osteoblastogenesis, osteoblast‐dependent osteoclastogenesis, and bone remodeling. J Cell Biol. 2006;172(1):115‐125. 16380437 10.1083/jcb.200507106 PMC2063539

[105] Roholl PJ , Blauw E , Zurcher C , Dormans JA , Theuns HM . Evidence for a diminished maturation of preosteoblasts into osteoblasts during aging in rats: an ultrastructural analysis. J Bone Miner Res. 1994;9(3):355‐366. 8191929 10.1002/jbmr.5650090310

[106] Farr JN , Xu M , Weivoda MM , et al. Targeting cellular senescence prevents age‐related bone loss in mice. Nat Med. 2017;23(9):1072‐1079. 28825716 10.1038/nm.4385 PMC5657592

[107] Bacic D , Lehir M , Biber J , Kaissling B , Murer H , Wagner CA . The renal Na+/phosphate cotransporter NaPi‐IIa is internalized via the receptor‐mediated endocytic route in response to parathyroid hormone. Kidney Int. 2006;69(3):495‐503. 16514432 10.1038/sj.ki.5000148

[108] Xu H , Uno JK , Inouye M , et al. Regulation of intestinal NaPi‐IIb cotransporter gene expression by estrogen. Am J Physiol Gastrointest Liver Physiol. 2003;285(6):G1317‐G1324. 12893629 10.1152/ajpgi.00172.2003

[109] Williams GR , Bassett JHD . Thyroid diseases and bone health. J Endocrinol Invest. 2018;41(1):99‐109. 10.1007/s40618-017-0753-4 PMC5754375 28853052

[110] Visser WE , Visser TJ , Peeters RP . Thyroid disorders in older adults. Endocrinol Metab Clin North Am. 2013;42(2):287‐303. 23702402 10.1016/j.ecl.2013.02.008

[111] Tian Y , Xu Y , Fu Q , He M . Parathyroid hormone regulates osteoblast differentiation in a Wnt/β‐catenin‐dependent manner. Mol Cell Biochem. 2011;355(1‐2):211‐216. 21533763 10.1007/s11010-011-0856-8

[112] Kousteni S , Bilezikian JP . The cell biology of parathyroid hormone in osteoblasts. Curr Osteoporos Rep. 2008;6(2):72‐76. 18778567 10.1007/s11914-008-0013-9

[113] Rao SD . Epidemiology of parathyroid disorders. Best Pract Res Clin Endocrinol Metab. 2018;32(6):773‐780. 30559041 10.1016/j.beem.2018.12.003

[114] Gentili C , Morelli S , de Boland AR . Characterization of PTH/PTHrP receptor in rat duodenum: effects of ageing. J Cell Biochem. 2003;88(6):1157‐1167. 12647298 10.1002/jcb.10472

[115] Cavedon V , Milanese C , Laginestra FG , et al. Bone and skeletal muscle changes in oldest‐old women: the role of physical inactivity. Aging Clin Exp Res. 2020;32(2):207‐214. 31535334 10.1007/s40520-019-01352-x

[116] Noble BS , Peet N , Stevens HY , et al. Mechanical loading: biphasic osteocyte survival and targeting of osteoclasts for bone destruction in rat cortical bone. Am J Physiol Cell Physiol. 2003;284(4):C934‐C943. 12477665 10.1152/ajpcell.00234.2002

[117] Weinbaum S , Cowin SC , Zeng Y . A model for the excitation of osteocytes by mechanical loading‐induced bone fluid shear stresses. J Biomech. 1994;27(3):339‐360. 8051194 10.1016/0021-9290(94)90010-8

[118] Li X , Han L , Nookaew I , et al. Stimulation of Piezo1 by mechanical signals promotes bone anabolism. Elife. 2019;8:e49631. 10.7554/eLife.49631 PMC6779475 31588901

[119] Yellowley CE , Li Z , Zhou Z , Jacobs CR , Donahue HJ . Functional gap junctions between osteocytic and osteoblastic cells. J Bone Miner Res. 2000;15(2):209‐217. 10703922 10.1359/jbmr.2000.15.2.209

[120] He D , Liu F , Cui S , et al. Mechanical load‐induced HS production by periodontal ligament stem cells activates M1 macrophages to promote bone remodeling and tooth movement via STAT1. Stem Cell Res Ther. 2020;11(1):112. 32169104 10.1186/s13287-020-01607-9 PMC7071778

[121] Tagliaferri C , Wittrant Y , Davicco M‐J , Walrand S , Coxam V . Muscle and bone, two interconnected tissues. Ageing Res Rev. 2015;21:55‐70. 25804855 10.1016/j.arr.2015.03.002

[122] Vainionpää A , Korpelainen R , Väänänen HK , Haapalahti J , Jämsä T , Leppäluoto J . Effect of impact exercise on bone metabolism. Osteoporos Int. 2009;20(10):1725‐1733. 19262975 10.1007/s00198-009-0881-6

[123] Zheng Q , Kernozek T , Daoud‐Gray A , Borer KT . Anabolic bone stimulus requires a pre‐exercise meal and 45‐minute walking impulse of suprathreshold speed‐enhanced momentum to prevent or mitigate postmenopausal osteoporosis within circadian constraints. Nutrients. 2021;13(11):3727. 10.3390/nu13113727 PMC8620686 34835982

[124] Rozenberg S , Bruyère O , Bergmann P , et al. How to manage osteoporosis before the age of 50. Maturitas. 2020;138:14‐25. 32631584 10.1016/j.maturitas.2020.05.004

[125] Ferrari SL , Deutsch S , Baudoin C , et al. LRP5 gene polymorphisms and idiopathic osteoporosis in men. Bone. 2005;37(6):770‐775. 16168727 10.1016/j.bone.2005.06.017

[126] Rouleau C , Malorie M , Collet C , et al. Diagnostic yield of bone fragility gene panel sequencing in children and young adults referred for idiopathic primary osteoporosis at a single regional reference centre. Bone Rep. 2022;16:101176. 35252483 10.1016/j.bonr.2022.101176 PMC8892094

[127] Rocha‐Braz MG , Ferraz‐de‐Souza B . Genetics of osteoporosis: searching for candidate genes for bone fragility. Arch Endocrinol Metab. 2016;60(4):391‐401. 27533615 10.1590/2359-3997000000178 PMC10118722

[128] Patsch JM , Kohler T , Berzlanovich A , et al. Trabecular bone microstructure and local gene expression in iliac crest biopsies of men with idiopathic osteoporosis. J Bone Miner Res. 2011;26(7):1584‐1592. 21308775 10.1002/jbmr.344

[129] Kocijan R , Muschitz C , Geiger E , et al. Circulating microRNA signatures in patients with idiopathic and postmenopausal osteoporosis and fragility fractures. J Clin Endocrinol Metab. 2016;101(11):4125‐4134. 27552543 10.1210/jc.2016-2365

[130] Feichtinger X , Muschitz C , Heimel P , et al. Bone‐related Circulating MicroRNAs miR‐29b‐3p, miR‐550a‐3p, and miR‐324‐3p and their association to bone microstructure and histomorphometry. Sci Rep. 2018;8(1):4867. 29559644 10.1038/s41598-018-22844-2 PMC5861059

[131] Rendina D , De Filippo G , Iannuzzo G , Abate V , Strazzullo P , Falchetti A . Idiopathic osteoporosis and nephrolithiasis: two sides of the same coin? Int J Mol Sci. 2020;21(21):8183. 10.3390/ijms21218183 PMC7662860 33142950

[132] Arrabal‐Polo MA , Cano‐García MdC , Canales BK , Arrabal‐Martín M. Calcium nephrolithiasis and bone demineralization: pathophysiology, diagnosis, and medical management. Curr Opin Urol. 2014;24(6):633‐638. 25188231 10.1097/MOU.0000000000000111

[133] Center JR , Lyles KW , Bliuc D . Bisphosphonates and lifespan. Bone. 2020;141:115566. 32745686 10.1016/j.bone.2020.115566

[134] Hu B , Zhang Y , Zhang G , et al. Research progress of bone‐targeted drug delivery system on metastatic bone tumors. J Control Release. 2022;350:377‐388. 36007681 10.1016/j.jconrel.2022.08.034

[135] Coxon FP , Thompson K , Roelofs AJ , Ebetino FH , Rogers MJ . Visualizing mineral binding and uptake of bisphosphonate by osteoclasts and non‐resorbing cells. Bone. 2008;42(5):848‐860. 18325866 10.1016/j.bone.2007.12.225

[136] Frith JC , Mönkkönen J , Auriola S , Mönkkönen H , Rogers MJ . The molecular mechanism of action of the antiresorptive and antiinflammatory drug clodronate: evidence for the formation in vivo of a metabolite that inhibits bone resorption and causes osteoclast and macrophage apoptosis. Arthritis Rheum. 2001;44(9):2201‐2210. 11592386 10.1002/1529-0131(200109)44:9<2201::aid-art374>3.0.co;2-e

[137] Luckman SP , Coxon FP , Ebetino FH , Russell RG , Rogers MJ . Heterocycle‐containing bisphosphonates cause apoptosis and inhibit bone resorption by preventing protein prenylation: evidence from structure‐activity relationships in J774 macrophages. J Bone Miner Res. 1998;13(11):1668‐1678. 9797474 10.1359/jbmr.1998.13.11.1668

[138] Rogers MJ , Mönkkönen J , Munoz MA . Molecular mechanisms of action of bisphosphonates and new insights into their effects outside the skeleton. Bone. 2020;139:115493. 32569873 10.1016/j.bone.2020.115493

[139] Seki K , Kaneko T , Kamimoto A , et al. Medication‐related osteonecrosis of the jaw after tooth extraction in patients receiving pharmaceutical treatment for osteoporosis: a retrospective cohort study. J Dent Sci. 2022;17(4):1619‐1625. 36299343 10.1016/j.jds.2022.03.014 PMC9588785

[140] Ji X , Pushalkar S , Li Y , Glickman R , Fleisher K , Saxena D . Antibiotic effects on bacterial profile in osteonecrosis of the jaw. Oral Dis. 2012;18(1):85‐95. 21883710 10.1111/j.1601-0825.2011.01848.x PMC3232327

[141] Beth‐Tasdogan NH , Mayer B , Hussein H , Zolk O , Peter J‐U . Interventions for managing medication‐related osteonecrosis of the jaw. Cochrane Database Syst Rev. 2022;7:CD012432. 35866376 10.1002/14651858.CD012432.pub3 PMC9309005

[142] Black DM , Condra K , Adams AL , Eastell R . Bisphosphonates and the risk of atypical femur fractures. Bone. 2022;156:116297. 34920168 10.1016/j.bone.2021.116297

[143] Pienkowski D , Wood CL , Malluche HH . Trabecular bone microcrack accumulation in patients treated with bisphosphonates for durations up to 16 years. J Orthop Res. 2022. 10.1002/jor.25441 PMC10039958 36163612

[144] Reid IR , Horne AM , Mihov B , et al. Anti‐fracture efficacy of zoledronate in subgroups of osteopenic postmenopausal women: secondary analysis of a randomized controlled trial. J Intern Med. 2019;286(2):221‐229. 30887607 10.1111/joim.12901

[145] Reid IR . Efficacy, effectiveness and side effects of medications used to prevent fractures. J Intern Med. 2015;277(6):690‐706. 25495429 10.1111/joim.12339

[146] Black DM , Rosen CJ . Clinical practice. Postmenopausal Osteoporosis. N Engl J Med. 2016;374(3):254‐262. 26789873 10.1056/NEJMcp1513724

[147] Hadjiargyrou M . Effects of bisphosphonates on appendicular fracture repair in rodents. Bone. 2022;164:116542. 36041726 10.1016/j.bone.2022.116542

[148] Palui R , Durgia H , Sahoo J , Naik D , Kamalanathan S . Timing of osteoporosis therapies following fracture: the current status. Ther Adv Endocrinol Metab. 2022;13:20420188221112904. 35899183 10.1177/20420188221112904 PMC9310203

[149] Marx D , Rahimnejad Yazdi A , Papini M , Towler M . A review of the latest insights into the mechanism of action of strontium in bone. Bone Rep. 2020;12:100273. 32395571 10.1016/j.bonr.2020.100273 PMC7210412

[150] Diepenhorst NA , Leach K , Keller AN , et al. Divergent effects of strontium and calcium‐sensing receptor positive allosteric modulators (calcimimetics) on human osteoclast activity. Br J Pharmacol. 2018;175(21):4095‐4108. 29714810 10.1111/bph.14344 PMC6177624

[151] Bonnelye E , Chabadel A , Saltel F , Jurdic P . Dual effect of strontium ranelate: stimulation of osteoblast differentiation and inhibition of osteoclast formation and resorption in vitro. Bone. 2008;42(1):129‐138. 17945546 10.1016/j.bone.2007.08.043

[152] Diepenhorst N , Rueda P , Cook AE , Pastoureau P , Sabatini M , Langmead CJ . G protein‐coupled receptors as anabolic drug targets in osteoporosis. Pharmacol Ther. 2018;184:1‐12. 29080701 10.1016/j.pharmthera.2017.10.015

[153] Wang D , Yan C , Zhou L , Fan X . Changes in BMP‐2 expression and mechanical properties during treatment of rats with osteoporotic hindlimb fracture with strontium ranelate. J Musculoskelet Neuronal Interact. 2020;20(1):136‐141. 32131378 PMC7104574

[154] Koukou OI , Pappas LD , Chloropoulou P , et al. The effect of strontium ranelate on fracture healing: an animal study. Biomed Res Int. 2020;2020:1085324. 33415138 10.1155/2020/1085324 PMC7768587

[155] Lavet C , Mabilleau G , Chappard D , Rizzoli R , Ammann P . Strontium ranelate stimulates trabecular bone formation in a rat tibial bone defect healing process. Osteoporos Int. 2017;28(12):3475‐3487. 28956091 10.1007/s00198-017-4156-3

[156] Wu Q , Hu L , Yan R , et al. Strontium‐incorporated bioceramic scaffolds for enhanced osteoporosis bone regeneration. Bone Res. 2022;10(1):55. 35999199 10.1038/s41413-022-00224-x PMC9399250

[157] Abdollahi Boraei SB , Nourmohammadi J , Sadat Mahdavi F , et al. Effect of SrR delivery in the biomarkers of bone regeneration during the in vitro degradation of HNT/GN coatings prepared by EPD. Colloids Surf B Biointerfaces. 2020;190:110944. 32155456 10.1016/j.colsurfb.2020.110944

[158] O'Donnell S , Cranney A , Wells GA , Adachi JD , Reginster JY . Strontium ranelate for preventing and treating postmenopausal osteoporosis. Cochrane Database Syst Rev. 2006;(4):CD005326. 10.1002/14651858.CD005326.pub3 PMC8092570 17054253

[159] Curtis EM , Cooper C , Harvey NC . Cardiovascular safety of calcium, magnesium and strontium: what does the evidence say? Aging Clin Exp Res. 2021;33(3):479‐494. 33565045 10.1007/s40520-021-01799-x PMC7943433

[160] Mendes MM , Botelho PB , Ribeiro H . Vitamin D and musculoskeletal health: outstanding aspects to be considered in the light of current evidence. Endocr Connect. 2022;11(10) 10.1530/EC-21-0596 PMC9578072 36048470

[161] Benn BS , Ajibade D , Porta A , et al. Active intestinal calcium transport in the absence of transient receptor potential vanilloid type 6 and calbindin‐D9k. Endocrinology. 2008;149(6):3196‐3205. 18325990 10.1210/en.2007-1655 PMC2408805

[162] Zhu K , Prince RL . Calcium and bone. Clin Biochem. 2012;45(12):936‐942. 22609892 10.1016/j.clinbiochem.2012.05.006

[163] Yamaguchi T , Chattopadhyay N , Kifor O , Butters RR , Sugimoto T , Brown EM . Mouse osteoblastic cell line (MC3T3‐E1) expresses extracellular calcium (Ca2+o)‐sensing receptor and its agonists stimulate chemotaxis and proliferation of MC3T3‐E1 cells. J Bone Miner Res. 1998;13(10):1530‐1538. 9783541 10.1359/jbmr.1998.13.10.1530

[164] Kanatani M , Sugimoto T , Kanzawa M , Yano S , Chihara K . High extracellular calcium inhibits osteoclast‐like cell formation by directly acting on the calcium‐sensing receptor existing in osteoclast precursor cells. Biochem Biophys Res Commun. 1999;261(1):144‐148. 10405337 10.1006/bbrc.1999.0932

[165] Xu J , Wang C , Han R , et al. Evidence of reciprocal regulation between the high extracellular calcium and RANKL signal transduction pathways in RAW cell derived osteoclasts. J Cell Physiol. 2005;202(2):554‐562. 15389575 10.1002/jcp.20159

[166] Deng J , Yang Y , He J , et al. Vitamin D receptor activated by vitamin D administration alleviates *Mycobacterium tuberculosis*‐induced bone destruction by inhibiting NFκB‐mediated aberrant osteoclastogenesis. FASEB J. 2021;35(6):e21543. 34046950 10.1096/fj.202100135R PMC12315972

[167] Hou Y‐C , Wu C‐C , Liao M‐T , et al. Role of nutritional vitamin D in osteoporosis treatment. Clin Chim Acta. 2018;484:179‐191. 29782843 10.1016/j.cca.2018.05.035

[168] Kim KJ , Kim MS , Hong N , et al. Cardiovascular risks associated with calcium supplementation in patients with osteoporosis: a nationwide cohort study. Eur Heart J Cardiovasc Pharmacother. 2022;8(6):568‐577. 34244740 10.1093/ehjcvp/pvab054

[169] Kahwati LC , Weber RP , Pan H , et al. Vitamin D, calcium, or combined supplementation for the primary prevention of fractures in community‐dwelling adults: evidence report and systematic review for the US preventive services task force. JAMA. 2018;319(15):1600‐1612.2967730810.1001/jama.2017.21640

[170] LeBoff MS , Chou SH , Ratliff KA , et al. Supplemental vitamin D and incident fractures in midlife and older adults. N Engl J Med. 2022;387(4):299‐309. 35939577 10.1056/NEJMoa2202106 PMC9716639

[171] Méndez‐Sánchez L , Clark P , Winzenberg TM , Tugwell P , Correa‐Burrows P , Costello R . Calcium and vitamin D for increasing bone mineral density in premenopausal women. Cochrane Database Syst Rev. 2023;1(1):CD012664. 36705288 10.1002/14651858.CD012664.pub2 PMC9881395

[172] Chakhtoura M , Bacha DS , Gharios C , et al. Vitamin D supplementation and fractures in adults: a systematic umbrella review of meta‐analyses of controlled trials. J Clin Endocrinol Metab. 2022;107(3):882‐898. 34687206 10.1210/clinem/dgab742 PMC8852203

[173] Yao P , Bennett D , Mafham M , et al. Vitamin D and calcium for the prevention of fracture: a systematic review and meta‐analysis. JAMA Netw Open. 2019;2(12):e1917789. 31860103 10.1001/jamanetworkopen.2019.17789 PMC6991219

[174] Stock M , Schett G . Vitamin K‐dependent proteins in skeletal development and disease. Int J Mol Sci. 2021;22(17):9328. 10.3390/ijms22179328 PMC8430550 34502245

[175] Ma M‐L , Ma Z‐J , He Y‐L , et al. Efficacy of vitamin K2 in the prevention and treatment of postmenopausal osteoporosis: a systematic review and meta‐analysis of randomized controlled trials. Front Public Health. 2022;10:979649. 36033779 10.3389/fpubh.2022.979649 PMC9403798

[176] Booth SL , Tucker KL , Chen H , et al. Dietary vitamin K intakes are associated with hip fracture but not with bone mineral density in elderly men and women. Am J Clin Nutr. 2000;71(5):1201‐1208. 10799384 10.1093/ajcn/71.5.1201

[177] Salma ASS , Karim S , Ibrahim IM, et al. Effect of vitamin K on bone mineral density and fracture risk in adults: systematic review and meta‐analysis. Biomedicines. 2022;10(5):1048. 10.3390/biomedicines10051048 PMC9138595 35625785

[178] Fusaro M , Cianciolo G , Brandi ML , et al. Vitamin K and Osteoporosis. Nutrients. 2020;12(12):3625. 10.3390/nu12123625 PMC7760385 33255760

[179] Wang K , Wu Q , Li Z , et al. Vitamin K intake and breast cancer incidence and death: results from a prospective cohort study. Clin Nutr. 2021;40(5):3370‐3378. 33277073 10.1016/j.clnu.2020.11.009

[180] Bus K , Szterk A . Relationship between structure and biological activity of various vitamin K forms. Foods. 2021;10(12):3136. 34945687 10.3390/foods10123136 PMC8701896

[181] Camacho PM , Petak SM , Binkley N , et al. American Association of Clinical Endocrinologists and American College of Endocrinology Clinical Practice guidelines for the diagnosis and treatment of postmenopausal osteoporosis—2016. Endocr Pract. 2016;22(suppl 4):1111‐1118. 10.4158/EP161435.ESGL 27643923

[182] Du X , Gao N , Song X . Bioadhesive polymer/lipid hybrid nanoparticles as oral delivery system of raloxifene with enhancive intestinal retention and bioavailability. Drug Deliv. 2021;28(1):252‐260. 33501870 10.1080/10717544.2021.1872742 PMC7850345

[183] Saini D , Fazil M , Ali MM , Baboota S , Ali J . Formulation, development and optimization of raloxifene‐loaded chitosan nanoparticles for treatment of osteoporosis. Drug Deliv. 2015;22(6):823‐836. 24725026 10.3109/10717544.2014.900153

[184] Ahmed OA , Badr‐Eldin SM . In situ misemgel as a multifunctional dual‐absorption platform for nasal delivery of raloxifene hydrochloride: formulation, characterization, and in vivo performance. Int J Nanomedicine. 2018;13:6325‐6335. 30349253 10.2147/IJN.S181587 PMC6188068

[185] Yang S‐J , Chang C‐H , Young T‐H , Wang C‐H , Tseng T‐H , Wang M‐L . Human serum albumin‐based nanoparticles alter raloxifene administration and improve bioavailability. Drug Deliv. 2022;29(1):2685‐2693. 35975329 10.1080/10717544.2022.2111479 PMC9387319

[186] Nagai N , Ogata F , Otake H , Nakazawa Y , Kawasaki N . Design of a transdermal formulation containing raloxifene nanoparticles for osteoporosis treatment. Int J Nanomedicine. 2018;13:5215‐5229. 30233182 10.2147/IJN.S173216 PMC6135211

[187] Zhou L , Poon CC‐W , Wong K‐Y , et al. Icariin ameliorates estrogen‐deficiency induced bone loss by enhancing IGF‐I signaling via its crosstalk with non‐genomic ERα signaling. Phytomedicine. 2021;82:153413. 33339654 10.1016/j.phymed.2020.153413

[188] Zhou L , Wong K‐Y , Yu W , et al. Selective estrogen receptor modulator‐like activities of Herba epimedii extract and its interactions with tamoxifen and raloxifene in bone cells and tissues. Front Pharmacol. 2020;11:571598. 33519435 10.3389/fphar.2020.571598 PMC7843570

[189] Wang K , Chen Y , Gao S , et al. Norlichexanthone purified from plant endophyte prevents postmenopausal osteoporosis by targeting ER to inhibit RANKL signaling. Acta Pharm Sin B. 2021;11(2):442‐455. 33643823 10.1016/j.apsb.2020.09.012 PMC7893202

[190] Seoud MA , Johnson J , Weed JC . Gynecologic tumors in tamoxifen‐treated women with breast cancer. Obstet Gynecol. 1993;82(2):165‐169. 8393156

[191] Maffei S , Guiducci L . Effect of ospemifene on densitometric and plasma bone metabolism biomarkers in postmenopausal women reporting vulvar and vaginal atrophy (VVA). J Clin Med. 2022;11(21) 10.3390/jcm11216316 PMC9655461 36362542

[192] Cummings SR , Ensrud K , Delmas PD , et al. Lasofoxifene in postmenopausal women with osteoporosis. N Engl J Med. 2010;362(8):686‐696. 20181970 10.1056/NEJMoa0808692

[193] Miyata J , Kasahara C , Asano T , et al. Orally available pyridinylpyrimidine derivatives as novel RANKL‐induced osteoclastogenesis inhibitors. Bioorg Med Chem Lett. 2012;22(17):5681‐5684. 22853997 10.1016/j.bmcl.2012.06.087

[194] Morikawa N , Kato Y , Takeshita N , Shimizu Y . Pharmacological characterization of AS2690168, a novel small molecule RANKL signal transduction inhibitor. Eur J Pharmacol. 2022;924:174941. 35398031 10.1016/j.ejphar.2022.174941

[195] Liu Z , Yin Y , Wang Z , et al. RANKL inhibition halts lesion progression and promotes bone remineralization in mice with fibrous dysplasia. Bone. 2022;156:116301. 34952228 10.1016/j.bone.2021.116301

[196] Nakai Y , Okamoto K , Terashima A , et al. Efficacy of an orally active small‐molecule inhibitor of RANKL in bone metastasis. Bone Res. 2019;7:1. 30622830 10.1038/s41413-018-0036-5 PMC6315020

[197] Melagraki G , Ntougkos E , Rinotas V , et al. Cheminformatics‐aided discovery of small‐molecule protein–protein interaction (PPI) dual inhibitors of tumor necrosis factor (TNF) and receptor activator of NF‐κB ligand (RANKL). PLoS Comput Biol. 2017;13(4):e1005372. 28426652 10.1371/journal.pcbi.1005372 PMC5398486

[198] Melagraki G , Ntougkos E , Papadopoulou D , et al. Discovery of plant‐origin natural product inhibitors of tumor necrosis factor (TNF) and receptor activator of NF‐κB ligand (RANKL). Front Pharmacol. 2018;9:800. 30090063 10.3389/fphar.2018.00800 PMC6068282

[199] Jiang M , Peng L , Yang K , et al. Development of small‐molecules targeting receptor activator of nuclear factor‐κB Ligand (RANKL)‐receptor activator of nuclear factor‐κB (RANK) protein‐protein interaction by structure‐based virtual screening and hit optimization. J Med Chem. 2019;62(11):5370‐5381. 31082234 10.1021/acs.jmedchem.8b02027

[200] Yang K , Li S , Wang T , et al. Development of an orally active small‐molecule inhibitor of receptor activator of nuclear factor‐κB ligand. J Med Chem. 2022;65(16):10992‐11009. 35960655 10.1021/acs.jmedchem.2c00081

[201] Chypre M , Madel M‐B , Chaloin O , Blin‐Wakkach C , Morice C , Mueller CG . Porphyrin derivatives inhibit the interaction between receptor activator of NF‐κB and its ligand. ChemMedChem. 2017;12(20):1697‐1702. 28885764 10.1002/cmdc.201700462

[202] He MM , Smith AS , Oslob JD , et al. Small‐molecule inhibition of TNF‐alpha. Science. 2005;310(5750):1022‐1025. 16284179 10.1126/science.1116304

[203] Rinotas V , Papakyriakou A , Violitzi F , et al. Discovery of small‐molecule inhibitors of receptor activator of nuclear factor‐κB Ligand with a superior therapeutic index. J Med Chem. 2020;63(20):12043‐12059. 32955874 10.1021/acs.jmedchem.0c01316

[204] Huang D , Zhao C , Li R , et al. Identification of a binding site on soluble RANKL that can be targeted to inhibit soluble RANK–RANKL interactions and treat osteoporosis. Nat Commun. 2022;13(1):5338. 36097003 10.1038/s41467-022-33006-4 PMC9468151

[205] Elango J , Bao B , Wu W . The hidden secrets of soluble RANKL in bone biology. Cytokine. 2021;144:155559. 33994070 10.1016/j.cyto.2021.155559

[206] Muthusamy K , Mohan S , Nagamani S , Kesavan C . Identification of novel small molecules that bind to the loop2 region of sclerostin—an in silico computational analysis. Physiol Res. 2016;65(5):871‐878. 27429110 10.33549/physiolres.933267

[207] Yooin W , Saenjum C , Ruangsuriya J , Jiranusornkul S . Discovery of potential sclerostin inhibitors from plants with loop2 region of sclerostin inhibition by interacting with residues outside Pro‐Asn‐Ala‐Ile‐Gly motif. J Biomol Struct Dyn. 2020;38(5):1272‐1282. 30907243 10.1080/07391102.2019.1599427

[208] Choi J , Lee K , Kang M , Lim S‐K , Tai No K . In silico discovery of quinoxaline derivatives as novel LRP5/6‐sclerostin interaction inhibitors. Bioorg Med Chem Lett. 2018;28(6):1116‐1121. 29486968 10.1016/j.bmcl.2018.01.050

[209] Kendler DL , Cosman F , Stad RK , Ferrari S . Denosumab in the treatment of osteoporosis: 10 years later: a narrative review. Adv Ther. 2022;39(1):58‐74. 34762286 10.1007/s12325-021-01936-y PMC8799550

[210] Kim AS , Girgis CM , McDonald MM . Osteoclast recycling and the rebound phenomenon following denosumab discontinuation. Curr Osteoporos Rep. 2022; 10.1007/s11914-022-00756-5 PMC9718877 36201122

[211] Ferrari S , Lewiecki EM , Butler PW , et al. Favorable skeletal benefit/risk of long‐term denosumab therapy: a virtual‐twin analysis of fractures prevented relative to skeletal safety events observed. Bone. 2020;134:115287. 32092479 10.1016/j.bone.2020.115287

[212] Morkos M , Mahrous P , Casagrande A , et al. Patterns of osteoporosis medications selection after drug holiday or continued therapy: a real‐world experience. Endocr Pract. 2022;28(10):1078‐1085. 35787466 10.1016/j.eprac.2022.06.011

[213] Ha J , Kim J , Jeong C , et al. Effect of follow‐up raloxifene therapy after denosumab discontinuation in postmenopausal women. Osteoporos Int. 2022;33(7):1591‐1599. 35376989 10.1007/s00198-022-06388-w PMC8978765

[214] Hong N , Shin S , Lee S , Kim KJ , Rhee Y . Raloxifene use after denosumab discontinuation partially attenuates bone loss in the lumbar spine in postmenopausal osteoporosis. Calcif Tissue Int. 2022;111(1):47‐55. 35226133 10.1007/s00223-022-00962-4

[215] Ko YJ , Sohn HM , Jang Y , et al. A novel modified RANKL variant can prevent osteoporosis by acting as a vaccine and an inhibitor. Clin Transl Med. 2021;11(3):e368. 33784004 10.1002/ctm2.368 PMC7967917

[216] Ko Y , Lee G , Kim B , Park M , Jang Y , Lim W . Modification of the RANKL–RANK‐binding site for the immunotherapeutic treatment of osteoporosis. Osteoporos Int. 2020;31(5):983‐993. 31863125 10.1007/s00198-019-05200-6

[217] Jiang Q , Zhang F , Zhu B , Zhang H , Han L , Liu X . Comparative efficacy between monoclonal antibodies and conventional drugs in postmenopausal women with osteoporosis: a network meta‐analysis. Ann Palliat Med. 2021;10(2):1693‐1702. 33302631 10.21037/apm-20-1294

[218] Dai Z , Fang P , Yan X , et al. Single Dose of SHR‐1222, a sclerostin monoclonal antibody, in healthy men and postmenopausal women with low bone mass: a randomized, double‐blind, placebo‐controlled, dose‐escalation, phase I study. Front Pharmacol. 2021;12:770073. 34744750 10.3389/fphar.2021.770073 PMC8564351

[219] Glorieux FH , Devogelaer J‐P , Durigova M , et al. BPS804 anti‐sclerostin antibody in adults with moderate osteogenesis imperfecta: results of a randomized phase 2a trial. J Bone Miner Res. 2017;32(7):1496‐1504. 28370407 10.1002/jbmr.3143

[220] Yu S , Li D , Zhang N , et al. Drug discovery of sclerostin inhibitors. Acta Pharm Sin B. 2022;12(5):2150‐2170. 35646527 10.1016/j.apsb.2022.01.012 PMC9136615

[221] Bovijn J , Krebs K , Chen C‐Y , et al. Evaluating the cardiovascular safety of sclerostin inhibition using evidence from meta‐analysis of clinical trials and human genetics. Sci Transl Med. 2020;12(549):eaay6570. 32581134 10.1126/scitranslmed.aay6570 PMC7116615

[222] Fixen C , Tunoa J . Romosozumab: a review of efficacy, safety, and cardiovascular risk. Curr Osteoporos Rep. 2021;19(1):15‐22. 33409990 10.1007/s11914-020-00652-w

[223] Estell EG , Rosen CJ . Emerging insights into the comparative effectiveness of anabolic therapies for osteoporosis. Nat Rev Endocrinol. 2021;17(1):31‐46. 33149262 10.1038/s41574-020-00426-5

[224] Florio M , Gunasekaran K , Stolina M , et al. A bispecific antibody targeting sclerostin and DKK‐1 promotes bone mass accrual and fracture repair. Nat Commun. 2016;7:11505. 27230681 10.1038/ncomms11505 PMC4894982

[225] Jiang H , Zhang Z , Yu Y , et al. Drug discovery of DKK1 inhibitors. Front Pharmacol. 2022;13:847387. 35355709 10.3389/fphar.2022.847387 PMC8959454

[226] Naot D , Musson DS , Cornish J . The activity of peptides of the calcitonin family in bone. Physiol Rev. 2019;99(1):781‐805. 30540227 10.1152/physrev.00066.2017

[227] Yazdani J , Khiavi RK , Ghavimi MA , Mortazavi A , Hagh EJ , Ahmadpour F . Calcitonin as an analgesic agent: review of mechanisms of action and clinical applications. Braz J Anesthesiol. 2019;69(6):594‐604. 31810524 10.1016/j.bjane.2019.08.003 PMC9391842

[228] Yu P , Liu Y , Xie J , Li J . Spatiotemporally controlled calcitonin delivery: long‐term and targeted therapy of skeletal diseases. J Control Release. 2021;338:486‐504. 34481022 10.1016/j.jconrel.2021.08.056

[229] Yu P , Liu Y , Jin R , et al. Thermosensitive polysaccharide hydrogel as a versatile platform for prolonged salmon calcitonin release and calcium regulation. ACS Biomater Sci Eng. 2020;6(7):4077‐4086. 33463337 10.1021/acsbiomaterials.0c00591

[230] Yu P , Xie J , Chen Y , et al. A thermo‐sensitive injectable hydroxypropyl chitin hydrogel for sustained salmon calcitonin release with enhanced osteogenesis and hypocalcemic effects. J Mater Chem B. 2020;8(2):270‐281. 31802093 10.1039/c9tb02049g

[231] Liu Y , Chen X , Li S , et al. Calcitonin‐loaded thermosensitive hydrogel for long‐term antiosteopenia therapy. ACS Appl Mater Interfaces. 2017;9(28):23428‐23440. 28640588 10.1021/acsami.7b05740

[232] Liu Y , Yu P , Peng X , et al. Hexapeptide‐conjugated calcitonin for targeted therapy of osteoporosis. J Control Release. 2019;304:39‐50. 31054990 10.1016/j.jconrel.2019.04.042

[233] Wang Z , Fu M , Wang Y , Meng Q , Guan Y , Zhang Y . Injectable carrier for zero‐order release of salmon calcitonin. ACS Biomater Sci Eng. 2020;6(1):485‐493. 33463212 10.1021/acsbiomaterials.9b01680

[234] Srinivasan A , Wong FK , Karponis D . Calcitonin: a useful old friend. J Musculoskelet Neuronal Interact. 2020;20(4):600‐609. 33265089 PMC7716677

[235] McLaughlin MB , Jialal I . Calcitonin . In: StatPearls. Treasure Island (FL): StatPearls Publishing; July 18, 2022.

[236] Rossini M , Adami G , Adami S , Viapiana O , Gatti D . Safety issues and adverse reactions with osteoporosis management. Expert Opin Drug Safety. 2016;15(3):321‐32. 10.1517/14740338.2016.1136287 26699669

[237] Bandeira L , Lewiecki EM , Bilezikian JP . Pharmacodynamics and pharmacokinetics of oral salmon calcitonin in the treatment of osteoporosis. Expert Opin Drug Metab Toxicol. 2016;12(6):681‐689. 27070719 10.1080/17425255.2016.1175436

[238] Chen T , Wang Y , Hao Z , Hu Y , Li J . Parathyroid hormone and its related peptides in bone metabolism. Biochem Pharmacol. 2021;192:114669. 34224692 10.1016/j.bcp.2021.114669

[239] Rutkovskiy A , Stensløkken K‐O , Vaage IJ . Osteoblast differentiation at a glance. Med Sci Monit Basic Res. 2016;22:95‐106. 27667570 10.12659/MSMBR.901142 PMC5040224

[240] Sun P , Wang M , Yin G‐Y . Endogenous parathyroid hormone (PTH) signals through osteoblasts via RANKL during fracture healing to affect osteoclasts. Biochem Biophys Res Commun. 2020;525(4):850‐856. 32169280 10.1016/j.bbrc.2020.02.177

[241] Hattersley G , Dean T , Corbin BA , Bahar H , Gardella TJ . Binding selectivity of abaloparatide for PTH‐Type‐1‐receptor conformations and effects on downstream signaling. Endocrinology. 2016;157(1):141‐149. 26562265 10.1210/en.2015-1726 PMC4701881

[242] Chandler H , Lanske B , Varela A , et al. Abaloparatide, a novel osteoanabolic PTHrP analog, increases cortical and trabecular bone mass and architecture in orchiectomized rats by increasing bone formation without increasing bone resorption. Bone. 2019;120:148‐155. 30343166 10.1016/j.bone.2018.10.012

[243] Sahbani K , Cardozo CP , Bauman WA , Tawfeek HA . Abaloparatide exhibits greater osteoanabolic response and higher cAMP stimulation and β‐arrestin recruitment than teriparatide. Physiol Rep. 2019;7(19):e14225. 31565870 10.14814/phy2.14225 PMC6766518

[244] Jolette J , Attalla B , Varela A , et al. Comparing the incidence of bone tumors in rats chronically exposed to the selective PTH type 1 receptor agonist abaloparatide or PTH(1‐34). Regul Toxicol Pharmacol. 2017;86:356‐365. 28389324 10.1016/j.yrtph.2017.04.001

[245] Kellier‐Steele N , Casso D , Anderson A , Oliveria SA , Motsko S . Assessing the incidence of osteosarcoma among teriparatide‐treated patients using linkage of commercial pharmacy and state cancer registry data, contributing to the removal of boxed warning and other labeling changes. Bone. 2022;160:116394. 35318162 10.1016/j.bone.2022.116394

[246] Gilsenan A , Midkiff K , Harris D , Kellier‐Steele N , McSorley D , Andrews EB . Teriparatide did not increase adult osteosarcoma incidence in a 15‐Year US postmarketing surveillance study. J Bone Miner Res. 2021;36(2):244‐251. 32990990 10.1002/jbmr.4188 PMC7988650

[247] Sim J , Kang G , Yang H , et al. Development of clinical weekly‐dose teriparatide acetate encapsulated dissolving microneedle patch for efficient treatment of osteoporosis. Polymers (Basel). 2022;14(19):4027. 36235975 10.3390/polym14194027 PMC9571303

[248] Subedi L , Pandey P , Kang SH , et al. Enhanced oral absorption of teriparatide with therapeutic potential for management of osteoporosis. J Control Release. 2022;349:502‐519. 35835400 10.1016/j.jconrel.2022.07.012

[249] An JM , Shahriar SMS , Hwang YH , et al. Oral delivery of parathyroid hormone using a triple‐padlock nanocarrier for osteoporosis an enterohepatic circulation pathway. ACS Appl Mater Interfaces. 2021;13(20):23314‐23327. 33587600 10.1021/acsami.0c22170

[250] Altaani BM , Almaaytah AM , Dadou S , Alkhamis K , Daradka MH , Hananeh W . Oral delivery of teriparatide using a nanoemulsion system: DESIGN, in vitro and in vivo evaluation. Pharm Res. 2020;37(4):80. 32253527 10.1007/s11095-020-02793-0

[251] Ganapathy A , Nieves JW , Keaveny TM , Cosman F . Effects of four‐year cyclic versus two‐year daily teriparatide treatment on volumetric bone density and bone strength in postmenopausal women with osteoporosis. Bone. 2023;167:116618. 36410666 10.1016/j.bone.2022.116618 PMC9822869

[252] Martin TJ , Seeman E . Abaloparatide is an anabolic, but does it spare resorption? J Bone Miner Res. 2017;32(1):11‐16. 27859635 10.1002/jbmr.3042

[253] Cheng X , Kinosaki M , Takami M , Choi Y , Zhang H , Murali R . Disabling of receptor activator of nuclear factor‐kappaB (RANK) receptor complex by novel osteoprotegerin‐like peptidomimetics restores bone loss in vivo. J Biol Chem. 2004;279(9):8269‐8277. 14679212 10.1074/jbc.M309690200

[254] Arai Y , Aoki K , Shimizu Y , et al. Peptide‐induced de novo bone formation after tooth extraction prevents alveolar bone loss in a murine tooth extraction model. Eur J Pharmacol. 2016;782:89‐97. 27118173 10.1016/j.ejphar.2016.04.049

[255] Uehara T , Mise‐Omata S , Matsui M , et al. Delivery of RANKL‐Binding Peptide OP3‐4 Promotes BMP‐2‐induced maxillary bone regeneration. J Dent Res. 2016;95(6):665‐672. 27006466 10.1177/0022034516633170

[256] Aoki K , Saito H , Itzstein C , et al. A TNF receptor loop peptide mimic blocks RANK ligand‐induced signaling, bone resorption, and bone loss. J Clin Invest. 2006;116(6):1525‐1534. 16680194 10.1172/JCI22513 PMC1448165

[257] Kou Y , Li C , Yang P , et al. The W9 peptide inhibits osteoclastogenesis and osteoclast activity by downregulating osteoclast autophagy and promoting osteoclast apoptosis. J Mol Histol. 2022;53(1):27‐38. 34664129 10.1007/s10735-021-10030-0

[258] Udagawa N , Koide M , Nakamura M , et al. Osteoclast differentiation by RANKL and OPG signaling pathways. J Bone Miner Metab. 2021;39(1):19‐26. 33079279 10.1007/s00774-020-01162-6

[259] Otsuki Y , Ii M , Moriwaki K , Okada M , Ueda K , Asahi M . W9 peptide enhanced osteogenic differentiation of human adipose‐derived stem cells. Biochem Biophys Res Commun. 2018;495(1):904‐910. 29154826 10.1016/j.bbrc.2017.11.056

[260] Ozaki Y , Koide M , Furuya Y , et al. Treatment of OPG‐deficient mice with WP9QY, a RANKL‐binding peptide, recovers alveolar bone loss by suppressing osteoclastogenesis and enhancing osteoblastogenesis. PLoS One. 2017;12(9):e0184904. 28937990 10.1371/journal.pone.0184904 PMC5609750

[261] Ta HM , Nguyen GTT , Jin HM , et al. Structure‐based development of a receptor activator of nuclear factor‐kappaB ligand (RANKL) inhibitor peptide and molecular basis for osteopetrosis. Proc Natl Acad Sci U S A. 2010;107(47):20281‐20286. 21059944 10.1073/pnas.1011686107 PMC2996688

[262] Hur J , Ghosh A , Kim K , et al. Design of a RANK‐mimetic peptide inhibitor of osteoclastogenesis with enhanced RANKL‐binding affinity. Mol Cells. 2016;39(4):316‐321. 26923188 10.14348/molcells.2016.2286 PMC4844938

[263] McConnell M , Shieh A . Polypharmacy in osteoporosis treatment. Clin Geriatr Med. 2022;38(4):715‐726. 36210087 10.1016/j.cger.2022.05.011

[264] Leder BZ , Tsai JN , Uihlein AV , et al. Denosumab and teriparatide transitions in postmenopausal osteoporosis (the DATA‐Switch study): extension of a randomised controlled trial. Lancet. 2015;386(9999):1147‐1155. 26144908 10.1016/S0140-6736(15)61120-5 PMC4620731

[265] Lu J , Hu D , Ma C , Shuai B . Advances in our understanding of the mechanism of action of drugs (including traditional Chinese medicines) for the intervention and treatment of osteoporosis. Front Pharmacol. 2022;13:938447. 35774616 10.3389/fphar.2022.938447 PMC9237325

[266] Tabatabaei‐Malazy O , Salari P , Khashayar P , Larijani B . New horizons in treatment of osteoporosis. Daru. 2017;25(1):2. 28173850 10.1186/s40199-017-0167-z PMC5297185

[267] Cosman F , Nieves JW , Dempster DW . Treatment sequence matters: anabolic and antiresorptive therapy for osteoporosis. J Bone Miner Res. 2017;32(2):198‐202. 27925287 10.1002/jbmr.3051

[268] Compston JE , McClung MR , Leslie WD . Osteoporosis. Lancet. 2019;393(10169):364‐376. 30696576 10.1016/S0140-6736(18)32112-3

[269] Zhang C , Song C . Combination therapy of PTH and antiresorptive drugs on osteoporosis: a review of treatment alternatives. Front Pharmacol. 2020;11:607017. 33584284 10.3389/fphar.2020.607017 PMC7874063

[270] Langdahl B . Treatment of postmenopausal osteoporosis with bone‐forming and antiresorptive treatments: combined and sequential approaches. Bone. 2020;139:115516. 32622871 10.1016/j.bone.2020.115516

[271] McClung MR . Using osteoporosis therapies in combination. Curr Osteoporos Rep. 2017;15(4):343‐352. 28667435 10.1007/s11914-017-0376-x

[272] Sun Y , Li Y , Li J , et al. Efficacy of the combination of teriparatide and denosumab in the treatment of postmenopausal osteoporosis: a meta‐analysis. Front Pharmacol. 2022;13:888208. 35685637 10.3389/fphar.2022.888208 PMC9170942

[273] Lou S , Lv H , Yin P , Li Z , Tang P , Wang Y . Combination therapy with parathyroid hormone analogs and antiresorptive agents for osteoporosis: a systematic review and meta‐analysis of randomized controlled trials. Osteoporos Int. 2019;30(1):59‐70. 30539271 10.1007/s00198-018-4790-4

[274] Black DM , Greenspan SL , Ensrud KE , et al. The effects of parathyroid hormone and alendronate alone or in combination in postmenopausal osteoporosis. N Engl J Med. 2003;349(13):1207‐1215. 14500804 10.1056/NEJMoa031975

[275] Jörg DJ , Fuertinger DH , Cherif A , et al. Modeling osteoporosis to design and optimize pharmacological therapies comprising multiple drug types. Elife. 2022;11:e76228. 35942681 10.7554/eLife.76228 PMC9363122

[276] Jin HJ , Bae YK , Kim M , et al. Comparative analysis of human mesenchymal stem cells from bone marrow, adipose tissue, and umbilical cord blood as sources of cell therapy. Int J Mol Sci. 2013;14(9):17986‐18001. 24005862 10.3390/ijms140917986 PMC3794764

[277] Arjmand B , Sarvari M , Alavi‐Moghadam S , et al. Prospect of stem cell therapy and regenerative medicine in osteoporosis. Front Endocrinol (Lausanne). 2020;11:430. 32719657 10.3389/fendo.2020.00430 PMC7347755

[278] Lu C‐H , Chen Y‐A , Ke C‐C , Liu R‐S . Mesenchymal stem cell‐derived extracellular vesicle: a promising alternative therapy for osteoporosis. Int J Mol Sci. 2021;22(23):12750. 34884554 10.3390/ijms222312750 PMC8657894

[279] Zhang C , Zhang W , Zhu D , et al. Nanoparticles functionalized with stem cell secretome and CXCR4‐overexpressing endothelial membrane for targeted osteoporosis therapy. J Nanobiotechnology. 2022;20(1):35. 35033095 10.1186/s12951-021-01231-6 PMC8760699

[280] Qi X , Zhang J , Yuan H , et al. Exosomes secreted by human‐induced pluripotent stem cell‐derived mesenchymal stem cells repair critical‐sized bone defects through enhanced angiogenesis and osteogenesis in osteoporotic rats. Int J Biol Sci. 2016;12(7):836‐849. 27313497 10.7150/ijbs.14809 PMC4910602

[281] An JH , Park H , Song JA , et al. Transplantation of human umbilical cord blood‐derived mesenchymal stem cells or their conditioned medium prevents bone loss in ovariectomized nude mice. Tissue Eng Part A. 2013;19(5‐6):685‐696. 23215868 10.1089/ten.tea.2012.0047 PMC3568969

[282] Lin L , He E , Wang H , et al. Intravenous transplantation of human hair follicle‐derived mesenchymal stem cells ameliorates trabecular bone loss in osteoporotic mice. Front Cell Dev Biol. 2022;10:814949. 35359450 10.3389/fcell.2022.814949 PMC8960386

[283] Wang W , Wang Y , Hu J , et al. Untargeted metabolomics reveal the protective effect of bone marrow mesenchymal stem cell transplantation against ovariectomy‐induced osteoporosis in mice. Cell Transplant. 2022;31:9636897221079745. 35225020 10.1177/09636897221079745 PMC8891838

[284] Wang W , Wang Y , Tang Z , et al. Mesenchymal stem cells prevent ovariectomy‐induced osteoporosis formation in mice through intraosseous vascular remodeling. Biochem Biophys Res Commun. 2021;582:64‐71. 34689107 10.1016/j.bbrc.2021.10.033

[285] Mei H , Li X , Wu Y , et al. Enhanced PDGFR/Wnt/β‐catenin activity of mesenchymal stem cells with high migration ability rescue bone loss of osteoporosis. Cell Signal. 2022;97:110394. 35753532 10.1016/j.cellsig.2022.110394

[286] Khosla S , Hofbauer LC . Osteoporosis treatment: recent developments and ongoing challenges. Lancet Diabetes Endocrinol. 2017;5(11):898‐907. 28689769 10.1016/S2213-8587(17)30188-2 PMC5798872

[287] Drake MT , Clarke BL , Oursler MJ , Khosla S . Cathepsin K inhibitors for osteoporosis: biology, potential clinical utility, and lessons learned. Endocr Rev. 2017;38(4):325‐350. 28651365 10.1210/er.2015-1114 PMC5546879

[288] McClung MR , O'Donoghue ML , Papapoulos SE , et al. Odanacatib for the treatment of postmenopausal osteoporosis: results of the LOFT multicentre, randomised, double‐blind, placebo‐controlled trial and LOFT Extension study. Lancet Diabetes Endocrinol. 2019;7(12):899‐911. 31676222 10.1016/S2213-8587(19)30346-8

[289] Hussain SM , Ebeling PR , Barker AL , Beilin LJ , Tonkin AM , McNeil JJ . Association of plasma high‐density lipoprotein cholesterol level with risk of fractures in healthy older adults. JAMA Cardiol. 2023;8(3):268‐272. 36652261 10.1001/jamacardio.2022.5124 PMC9857824

[290] Tobias DK , Luttmann‐Gibson H , Mora S , et al. Association of body weight with response to vitamin d supplementation and metabolism. JAMA Netw Open. 2023;6(1):e2250681. 36648947 10.1001/jamanetworkopen.2022.50681 PMC9856931

[291] Reid IR , Billington EO . Drug therapy for osteoporosis in older adults. Lancet. 2022;399(10329):1080‐1092. 35279261 10.1016/S0140-6736(21)02646-5

